# Determining the genetic basis of anthracycline-cardiotoxicity by molecular response QTL mapping in induced cardiomyocytes

**DOI:** 10.7554/eLife.33480

**Published:** 2018-05-08

**Authors:** David A Knowles, Courtney K Burrows, John D Blischak, Kristen M Patterson, Daniel J Serie, Nadine Norton, Carole Ober, Jonathan K Pritchard, Yoav Gilad

**Affiliations:** 1 Department of GeneticsStanford UniversityStanfordUnited States; 2 Department of RadiologyStanford UniversityStanfordUnited States; 3 Department of Human GeneticsUniversity of ChicagoChicagoUnited States; 4 Department of Health Sciences ResearchMayo ClinicJacksonvilleUnited States; 5 Department of Cancer BiologyMayo ClinicJacksonvilleUnited States; 6 Department of BiologyStanford UniversityStanfordUnited States; 7 Howard Hughes Medical InstituteStanford UniversityStanfordUnited States; 8 Department of MedicineUniversity of ChicagoChicagoUnited States; Oxford UniversityUnited Kingdom

**Keywords:** anthracycline-induced toxicity, response expression QTL, iPSC-derived differentiated cells, Doxorubicin, oxidative damage, Human

## Abstract

Anthracycline-induced cardiotoxicity (ACT) is a key limiting factor in setting optimal chemotherapy regimes, with almost half of patients expected to develop congestive heart failure given high doses. However, the genetic basis of sensitivity to anthracyclines remains unclear. We created a panel of iPSC-derived cardiomyocytes from 45 individuals and performed RNA-seq after 24 hr exposure to varying doxorubicin dosages. The transcriptomic response is substantial: the majority of genes are differentially expressed and over 6000 genes show evidence of differential splicing, the later driven by reduced splicing fidelity in the presence of doxorubicin. We show that inter-individual variation in transcriptional response is predictive of in vitro cell damage, which in turn is associated with in vivo ACT risk. We detect 447 response-expression quantitative trait loci (QTLs) and 42 response-splicing QTLs, which are enriched in lower ACT GWAS p-values, supporting the in vivo relevance of our map of genetic regulation of cellular response to anthracyclines.

## eLife digest

Many cancers, including leukaemia, lymphoma and breast cancer, are treated with potent chemotherapy drugs such as anthracyclines. However, anthracyclines have strong side effects known as anthracycline cardiotoxicity, which affect the health of the heart. Almost half of the patients given high doses of anthracyclines develop chronic heart failure.

While anthracycline cardiotoxicity is very common, people’s genes may contribute to how sensitive they are to these drugs but it is not understood which genes can cause this effect. Previous studies using only a small number of participants have not been able to pin down the genetic factors that make some patients respond well to anthracyclines, and others prone to developing heart failure when taking these drugs.

To find out which genes affect anthracycline cardiotoxicity, Knowles, Burrows et al. transformed blood cells from 45 individuals into stem cells, which were then developed into heart muscle cells. Then, the activity of genes was analyzed by measuring the amount of RNA (the template molecules used to make proteins) produced by those genes.

After the cells had been exposed for 24 hours to the anthracycline drug doxorubicin, hundreds of gene activity differences could be found in the heart muscle cells between individuals. Some of these differences were linked to poorer health of the cells after treatment with the drug. As a result, a number of genetic variants that could predispose patients to the side effects of doxorubicin were discovered. The experiments also revealed how doxorubicin disrupts an important process that separates ‘junk’ parts of the RNA from the parts that are used as a template for proteins.

Being able to predict who is likely to be sensitive to drugs such as doxorubicin could help doctors to tailor chemotherapy treatments more effectively, minimising the risk of heart failure. In future, larger studies could lead to accurate predictions of a patient’s response to a particular chemotherapy drug to personalize their cancer treatment.

## Introduction

Anthracyclines, including the prototypical doxorubicin, continue to be used as chemotherapeutic agents treating a wide range of cancers, particularly leukemia, lymphoma, multiple myeloma, breast cancer, and sarcoma. A well-known side-effect of doxorubicin treatment is anthracycline-induced cardiotoxicity (ACT). For some patients ACT manifests as an asymptomatic reduction in cardiac function, as measured by left ventricular ejection fraction (LVEF), but in more extreme cases ACT can lead to congestive heart failure (CHF). The risk of CHF is dosage-dependent: an early study (Von Hoff et al., 1979) estimated 3% of patients at 400 mg/m2, 7% of patients at 550 mg/m2, and 18% of patients at 700 mg/m2 develop CHF, where a more recent study puts these numbers at 5%, 26% and 48% respectively (Swain et al., 2003). Reduced LVEF shows a similar dosage-dependent pattern, but is not fully predictive of CHF.

Perhaps most daunting for patients is that CHF can occur years after treatment: out of 1807 cancer survivors followed for 7 years in a recent survey a third died of heart diseases compared to 51% of cancer recurrence (Vejpongsa and Yeh, 2014).

Various candidate gene studies have attempted to find genetic determinants of ACT, but are plagued by small sample sizes and unclear endpoint definitions, resulting in limited replication between studies. Two ACT genome-wide association studies (GWAS) have been published (Aminkeng et al., 2015; Schneider et al., 2017). While neither found genome-wide significant associations using their discovery cohorts, both found one variant that they were able to replicate in independent cohorts.

A nonsynonymous coding variant, rs2229774, in *RARG* (retinoic acid receptor γ) was found to be associated with pediatric ACT using a Canadian European discovery cohort of 280 patients (Aminkeng et al., 2015), and replicated in both a European (p=0.004) and non-European cohort ($p=1\times 10^{-4}$). Modest signal (p=0.076) supporting rs2229774’s association with ACT was also reported in a recent study primarily focused on trastuzumab-related cardiotoxicity (Serie et al., 2017). *RARG* negative cell lines have reduced retinoic acid response element (RAREs) activity and reduced suppression of *Top2b* (Aminkeng et al., 2015), which has been proposed as a mediator of ACT.

In a different study, a GWAS in 845 patients with European-ancestry from a large adjuvant breast cancer clinical trial, 51 of whom developed CHF, found no variants at genome-wide significance levels (Schneider et al., 2017). However, one of the most promising variants, rs28714259 ($p=9\times 10^{-6}$ in discovery cohort), was genotyped in two further cohorts and showed modest replication (p=0.04,0.018). rs28714259 falls in a glucocorticoid receptor protein binding peak, which may play a role in cardiac development.

An exciting approach to studying complex phenotypes, including disease, in human is to use induced pluripotent stem cells (iPSC) and derived differentiated cells as in vitro model systems. Work by us and others has demonstrated that iPSCs and iPSC-derived cell-types are powerful model systems for understanding cell-type specific genetic regulation of transcription (Thomas et al., 2015; Burrows et al., 2016; Banovich et al., 2018; Kilpinen et al., 2017; Alasoo et al., 2017), but it is less established whether these systems can be used to model the interplay of genetic and environmental factors in disease progression. Encouragingly, the response of iPSC-derived cardiomyocytes (ICs) to doxorubicin was recently extensively characterized (Burridge et al., 2016). ICs derived from four individuals who developed ACT after doxorubicin treatment (‘DOXTOX’ group) and four who did not (‘DOX’ group), showed clear differences in viability (via apoptosis), metabolism, DNA damage, oxidative stress and mitochondrial function when exposed to doxorubicin. These observations suggest that ICs recapitulate in vivo inter-individual differences in doxorubicin sensitivity. Gene expression response differences between the DOX and DOXTOX groups were found using RNA-sequencing data, but the sample size was insufficient (RNA-seq was generated for only three individuals in each group) to attempt mapping of genetic variants that might explain the observed functional differences between individuals.

Here we used a panel of iPSC-derived cardiomyocytes from 45 individuals, exposed to five different drug concentrations, to map the genetic basis of inter-individual differences in doxorubicin-sensitivity. We find hundreds of genetics variants that modulate the transcriptomic response, including 42 that act on alternative splicing. We show that the IC transcriptomic response predicts cardiac troponin levels in culture (indicative of cell lysis) in these cell-lines, and that troponin level is itself predictive of ACT. Finally we demonstrate that the mapped genetic variants show significant enrichment in lower ACT GWAS p-values.

## Results

### Measuring transcriptomic response to doxorubicin exposure

**Figure 1.**
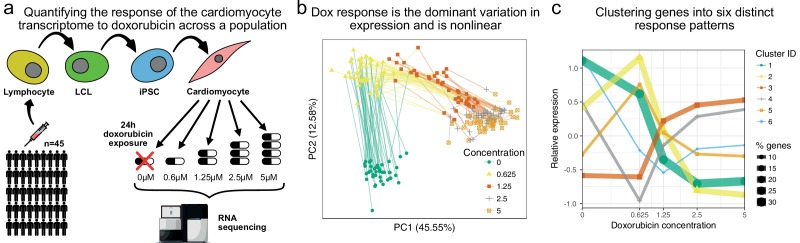
The transcriptomic response of cardiomyocytes to doxorubicin is substantial. (**a**) Cardiomyocytes were derived from lymphoblastoid cell lines (LCLs) of 45 Hutterite individuals, followed by exposure to differing concentrations of doxorubicin and RNA-sequencing. (**b**) PCA of gene expression levels across samples reveals that doxorubicin concentration explains more variance than inter-individual differences, and that the response is non-linear with respect to concentration. Lines connect samples from the same individual. (**c**) A probabilistic mixture model uncovers six distinct patterns of response across genes.

We generated iPSC-derived cardiomyocytes (ICs) for 45 Hutterite individuals (Figure 1a). iPSC quality was confirmed using qPCR (Figure 1—figure supplement 1), global gene expression profiling (Figure 1—figure supplement 2), the embryoid body test (Supplementary Data), and EBV integration analysis (Figure 1—figure supplement 3, Figure 1—figure supplement 4). Cardiomyocyte identity was confirmed by FACS for cardiac troponin I and T, with mean purity (72 ±12)% (Figure 1—figure supplement 5). We exposed all 45 IC lines to doxorubicin at five different concentrations for 24 hr, after which samples were processed for RNA-sequencing. We obtained sufficient read depth (10M exonic reads) for downstream analysis for 217 of the 5×45=225 individual-concentration pairs, and confirmed sample identity by calling exonic SNPs (see Methods). We observed a strong gene regulatory response to doxorubicin across all concentrations, with 98% (12038/12317) of quantifiable genes (5% FDR) showing differential expression across the different treatment concentrations. Our data shows excellent concordance with the smaller RNA-seq dataset of (Burridge et al., 2016) (Figure 1—figure supplement 6). Principal component analysis (PCA, Figure 1b) confirms that the main variation in the data is driven by doxorubicin concentration and that the effect of concentration on gene expression is nonlinear. For some individuals the expression data following doxorubicin treatment with 1.25μM is closer to the data from treatment with 0.625μM, whereas for others it is closer to data from treatment with 2.5μM. This general pattern provides the first indication in our data that that there is systematic variation in how different individuals respond to doxorubicin exposure. Since the majority of genes appear responsive to doxorubicin we clustered genes into six distinct response patterns using a mixture model approach (Figure 1c, see Materials and methods). From largest to smallest, these clusters represent genes that, through the gradient from low to high concentration treatments, are (1) down regulated (2) initially up-regulated, then further down-regulated (3) up-regulated (4) down-regulated only at lower dosages (5) up-regulated only at lower dosages (6) down-regulated then partially recover (Supplementary file 1). Gene set enrichments (Figure 1—figure supplement 7, Supplementary file 2) for the up-regulated cluster include metabolic, mitochrondrial and extracellular processes, as well as known doxorubicin response genes in breast cancer cell lines from (Graessmann et al., 2007) (647 overlapping genes of 1090 in term, hypergeometric $p=2\times 10^{-26}$). The down-regulated cluster shares genes with those down-regulated in response to UV light, which, like doxorubicin, causes DNA-damage (413 overlapping genes of 470 in term, hypergeometric $p=3\times 10^{-48}$). Targets of p53, a transcription factor that responds to DNA damage, are overrepresented in clusters 2 and 5; these clusters involve up-regulation at low concentrations (0.625μM) but down-regulation at higher concentrations (486 overlapping genes out of 1057 in term, hypergeometric $p=2\times 10^{-39}$). Promoter analysis (Figure 1—figure supplement 8, Supplementary file 3) revealed 21, 45, and 6 significantly enriched transcription factor (TF) binding motifs for clusters 1, 2 and 3 respectively (and none for cluster 4–6). Examples include binding sites for *ZNF143*, a TF that promotes *GPX1* activity and protects cells from oxidative damage during mitochondrial respiratory dysfunction (Lu et al., 2012), which is enriched in cluster 1 (down regulation w/dox, 318 overlapping genes out of 3555 *ZNF143* targets, hypergeometric $p=10^{-8}$); *RONIN*, a regulator of mitochrondrial development and function (Poché et al., 2016), which is enriched in clusters 1 and 2 (217 and 210 overlapping genes out of 2295 targets, $p=10^{-7}$ and $10^{-4}$ respectively); and *MEF2*, myocyte enhancer factor 2, involved in regulating muscle development, stress-response and p38-mediated apoptosis (Zarubin and Han, 2005), enriched in cluster 4 (32 overlapping genes out of 741 targets, hypergeometric $p=10^{-3}$).

**Figure 1—figure supplement 1.**
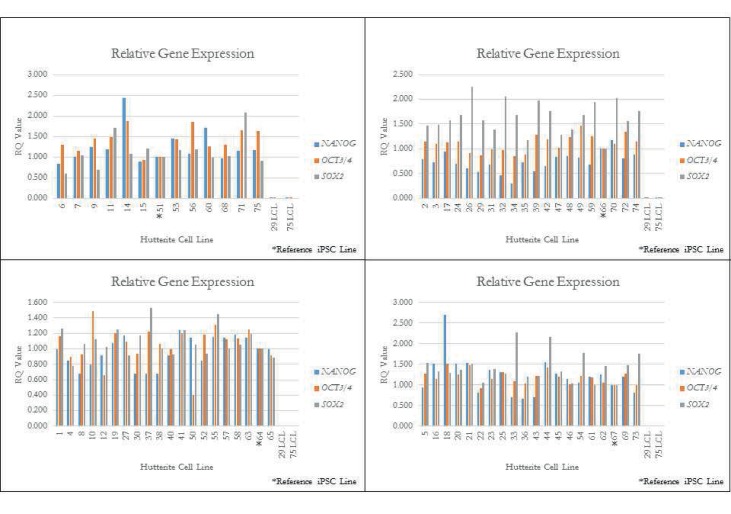
qPCR for key pluripotency genes for a subset of iPSCs. qPCR quality control of iPSC lines. Quantitative PCR (qPCR) of pluripotency genes *OCT3/4*, *NANOG*, and *SOX2* on randomly selected Hutterite iPSC lines (denoted by *). Relative expression is the RQ value with respect to *GAPDH* expression, with error bars representing the calculated min and max RQ value. All iPSC lines show endogenous expression of these pluripotency genes. Two original LCL lines are included as control and show little or no expression of pluripotency genes.

**Figure 1—figure supplement 2.**
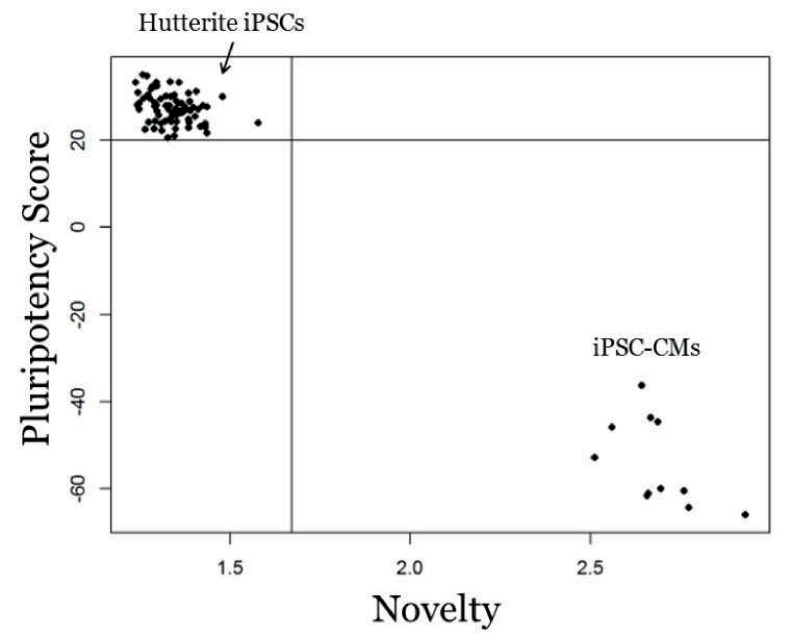
Genome-wide expression analysis of iPSCs using PluriTest. PluriTest quality control of iPSCs. PluriTest pluriscore and novelty results shows all iPSC samples fall within the pluripotent and novelty threshold (black lines). All cell lines received a pluripotency score above the pluripotency classification threshold of 20 (average = 27.38) and showed low novelty scores below the threshold of 1.67 (average = 1.34). As a comparison, a subset of the iPSC-cardiomyocytes fall outside of the pluripotency classification for both pluriscore and novelty.

**Figure 1—figure supplement 3.**
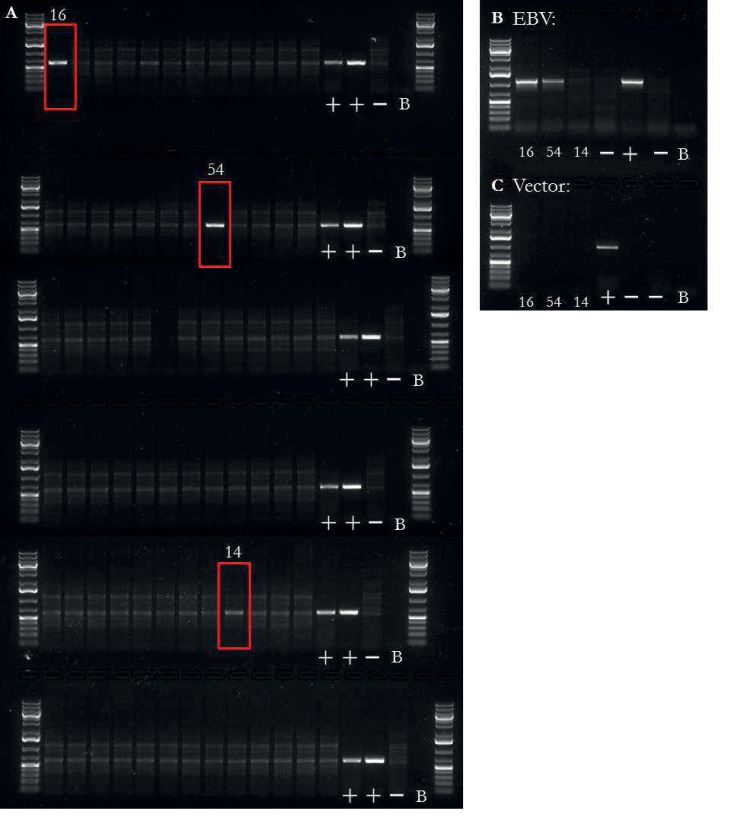
EBV integration analysis of iPSCs. EBV presence/absence quality control of iPSCs. (**a**) PCR on DNA for presence or absence of EBV, both integrated and non-integrated (reprogramming vector based). Two iPSC lines had obvious presence of EBV (16, 54) and one was unclear (14). Additional banding in the images was due to RNA in the sample. The three samples, highlighted by a red box, were taken forward for two additional PCRs. First, the three samples were tested for EBV based on the presence of the LMP-2A sequence (**b**); an EBV gene not found on the reprogramming vector), of which lines 16 and 54 were positive. Lastly, the three samples were tested for the presence of the reprogramming vectors (**c**), of which no samples were positive. Controls were ordered as followed for all PCRs: mixture of chimp and human fibroblast DNA at 10 ng/μL with all reprogramming plasmids at 0.02 pg/μL), mixture of two LCL lines DNA at 10 ng/μL, mixture of human and chimp fibroblast DNA at 10 ng/μL without any plasmid, and no sample blank.

**Figure 1—figure supplement 4.**
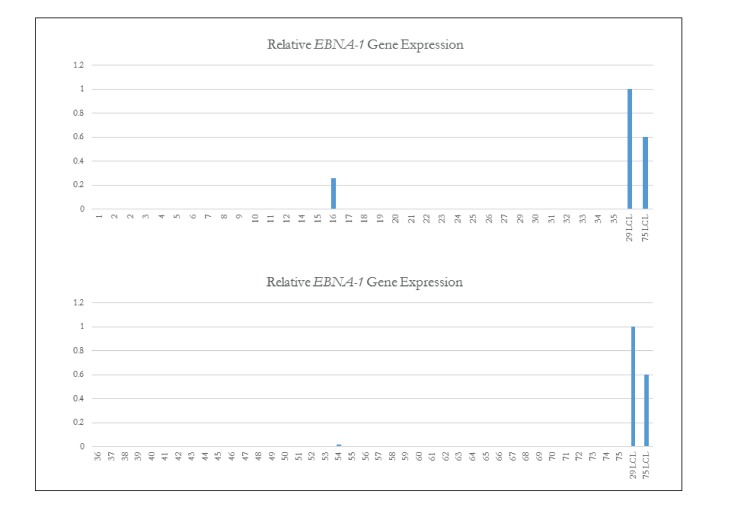
qPCR of *EBNA-1 to assess EBV status in iPSCs.* Quantitative PCR of EBNA-1 quality control of iPSC Lines. Quantitative PCR (qPCR) of EBV-required gene EBNA-1. Lines 16 and 54 which both showed the presence of genomic integrations of EBV (as shown in Fig. Figure 1—figure supplement 3) are the only iPSC lines that have EBNA-1 expression. Line 14 is again confirmed as not having EBV.

**Figure 1—figure supplement 5.**
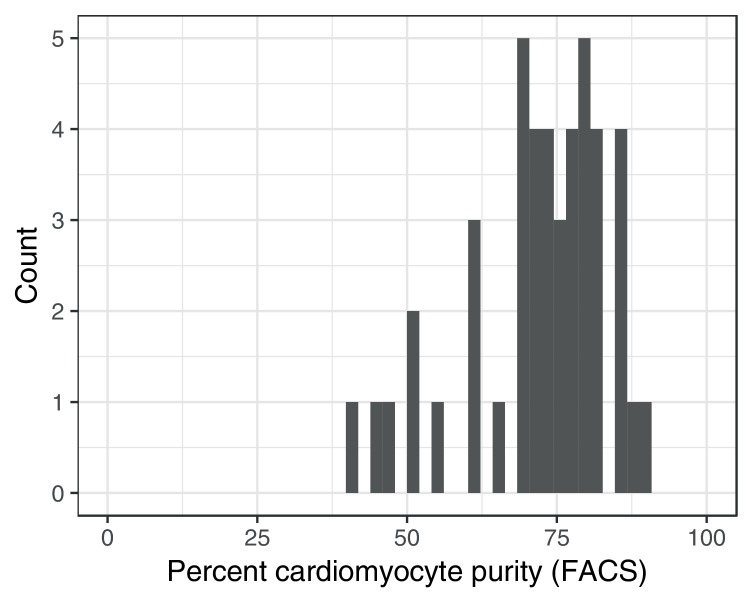
Fluorescence activated cell sorting (FACS) purity estimates for iPSC-derived cardiomyocytes.

**Figure 1—figure supplement 6.**
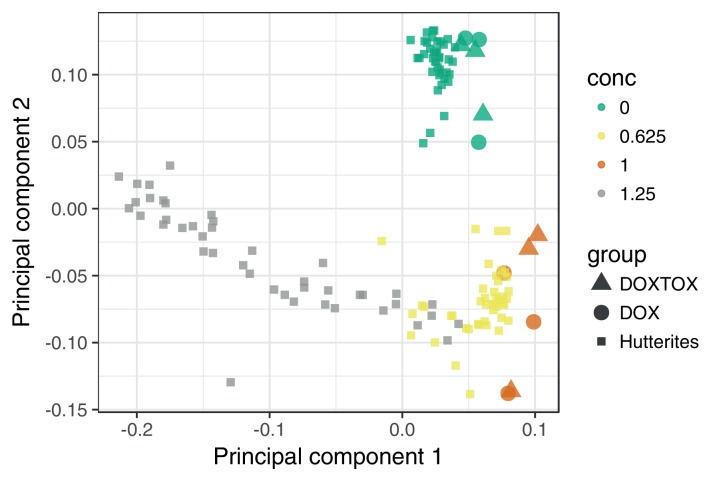
Our expression data is concordant with an existing small RNA-seqdataset (Burridge et al., 2016). DOXTOX and DOX correspond to samples from patients that did and did not develop ACT after doxorubicin chemotherapy respectively. We additionally see that the transcriptional response at higher concentrations cannot be extrapolated from that at lower concentrations. Higher concentrations are not shown since including these compresses the first PC obscuring the relevant variation.

**Figure 1—figure supplement 7.**
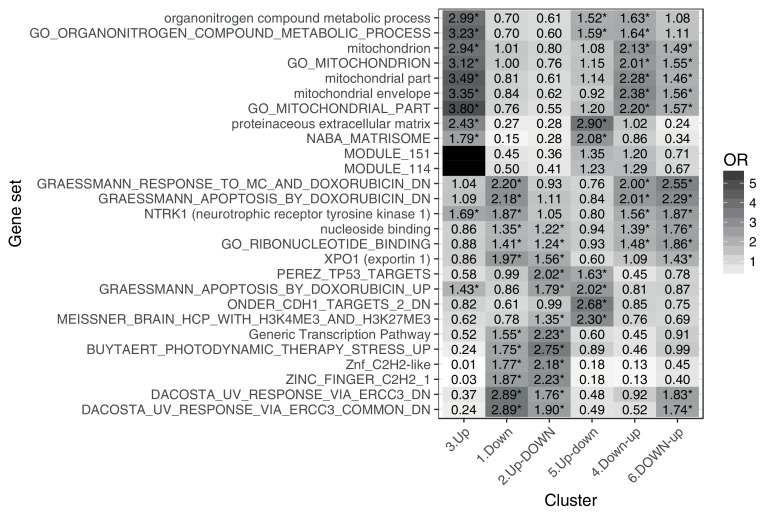
Gene set enrichment analysis of genes in each response cluster confirms expected patterns: metabolic, mitochrondrial and DNA damage processes, as well as existing doxorubicin response genes.

**Figure 1—figure supplement 8.**
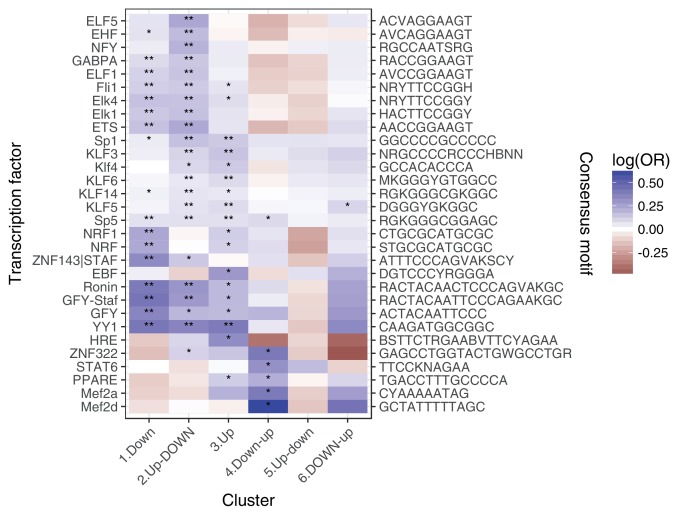
Enrichment of transcription factor binding motifs for each response pattern, using HOMER. ** denotes *q <* 0:05, * denotes *q <* 0:5.

### Mapping variants modulating doxorubicin response

**Figure 2.**
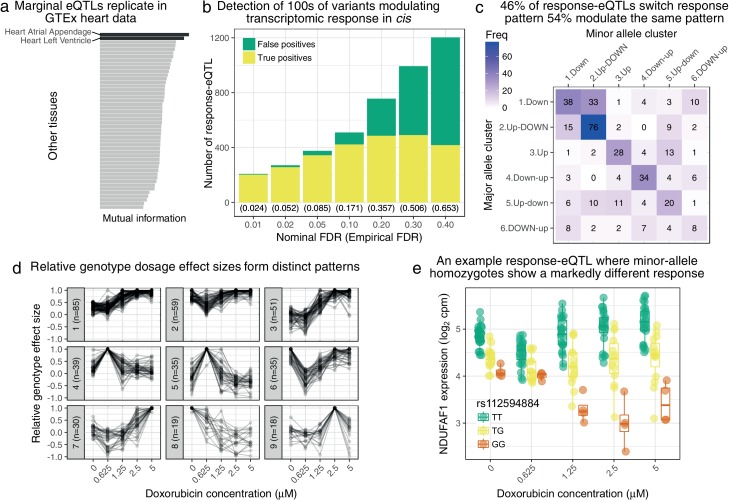
Genetic variation regulates the transcriptomic response to doxorubicin exposure. (**a**) Marginal eQTLs show strong replication in GTEx heart data, and lower replication in other tissues (LCL = lymphoblastoid cell line). (**b**) We detect 100 s of response-eQTLs (reQTLs): variants that modulate response to doxorubicin. The false positive rate (FPR) is estimated using a parametric bootstrap. (**c**) We developed a statistical method to assign the major and minor allele response to one of the six clusters from Figure 1c. The strongest 46% of detected reQTLs result in a discretely different response, whereas the remainder only modulate the response. (**d**) For significant reQTLs we calculated relative genotype effect sizes by dividing the fitted effect size at each concentration by the (signed) effect size with the largest absolute value. K-means clustering of these effect size profiles reveals distinct patterns, the most common being a small reduction in absolute effect size from 0 to 0.625μM followed by the largest effects being at the highest concentrations. (**e**) An example response-eQTL where rs112594884 regulates the response of the mitochondrial complex I chaperone NDUFAF1. Under the major (T) allele we see moderate down-regulation at 0.625μM followed by up-regulation at higher concentrations. Under the minor (G) allele, there is little change at 0.625μM followed by substantial down-regulation. Since the genotype effects are reduced at 0.625μM and largest at high concentrations this reQTL is assigned to cluster 1 of panel d.

We next sought to map single nucleotide polymorphisms (SNPs) that modulate the observed inter-individual transcriptomic response to doxorubicin, leveraging available genetic variation across the 45 individuals (Livne et al., 2015). We developed a linear mixed model approach, called suez, that extends the PANAMA framework (Fusi et al., 2012) to account for relatedness amongst individuals, repeat measurements, multiple conditions and latent confounding. Testing SNPs within 1 Mb of the transcription start site (TSS), 518 genes have a variant with a detectable marginal effect on expression (5% FDR, Supplementary file 4). Using a mutual information approach (see Methods) which, unlike a naive replication analysis (Figure 2—figure supplement 1), controls for differential power across GTEx tissues, we find our expression quantitative trait loci (eQTLs) show stronger overlap with the two heart tissues than any other GTEx tissue (Figure 2a, Figure 2—figure supplement 2). Remarkably, even with our moderate number of individuals, we are able to detect many response-eQTLs (reQTLs), i.e. variants that modulate (directly or indirectly) transcriptomic response to doxorubicin. We found reQTLs for 376 genes at a nominal 5% FDR (Supplementary file 5), which we estimate using a parametric bootstrap corresponds to a true FDR of 8.5% (Figure 2b). We explored leveraging allele specific expression (ASE) extending our previous work (Knowles et al., 2017; van de Geijn et al., 2015). We fit a beta-binomial generalized linear model (GLM) where the response variable corresponds to alternative vs reference read counts and the independent variable is the heterozygosity of the test regulatory eSNP. We found it impractical to directly relate effect sizes in the total expression and ASE models so we instead combined likelihood ratios from the beta-binomial GLM and suez likelihood into a single test statistic. This approach yielded 447 reQTLs at 5% FDR (Supplementary file 6), an increase of 19% over using total expression alone. We hypothesize that this relatively modest increase in power is due to a) suez already being reasonably well powered in this direct perturbation setting and b) the somewhat low sequencing depth of our samples.

**Figure 2—figure supplement 1.**
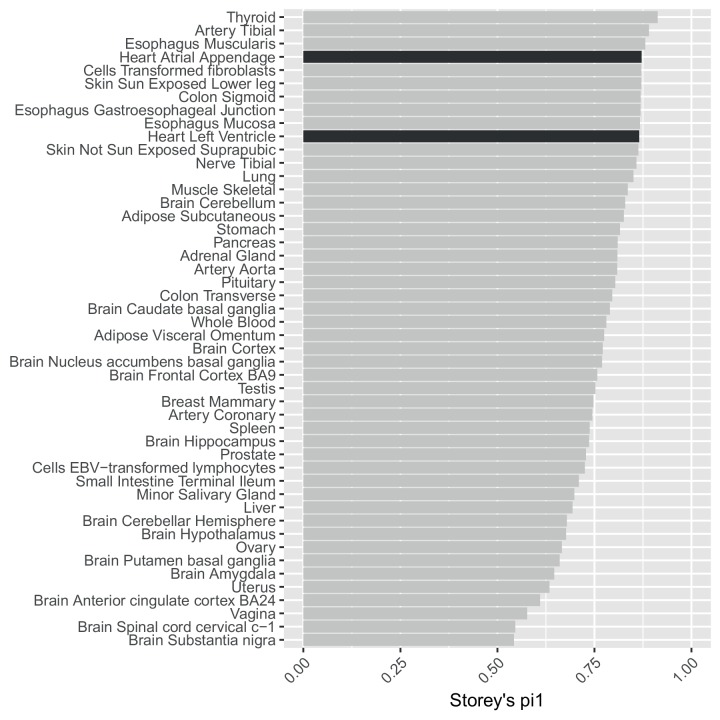
Using $\pi_{1}$ statistics to assess replication is confounded by different sample size/power across GTEx tissues. Storey’s $\pi_{1}$ values to estimate the replication in GTEx tissues of SNP-gene eQTL pairs significant in our data at $p<10^{-5}$.This analysis is confounded by variable power across the GTEx tissues.

**Figure 2—figure supplement 2.**
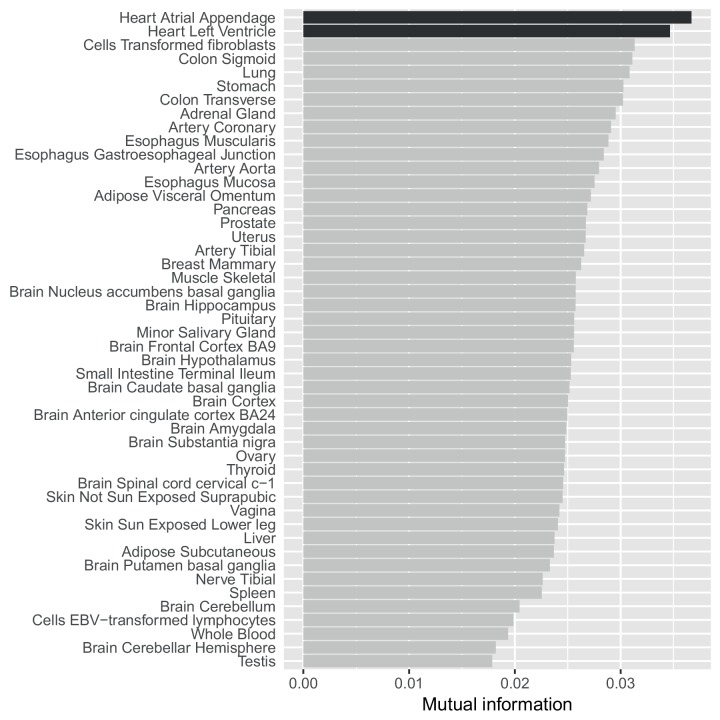
A mutual information approach to control for different sample sizes/power across GTEx tissues. Estimates of the mutual information between whether SNP-gene pairs are associated in our data compared to all 48 GTEx tissues. Unlike a simple replication analysis our approach controls for differential power across the GTEx tissues (see Materials and methods) and shows that our eQTLs agree most strongly with eQTLs from the two heart tissues.

**Figure 2—figure supplement 3.**
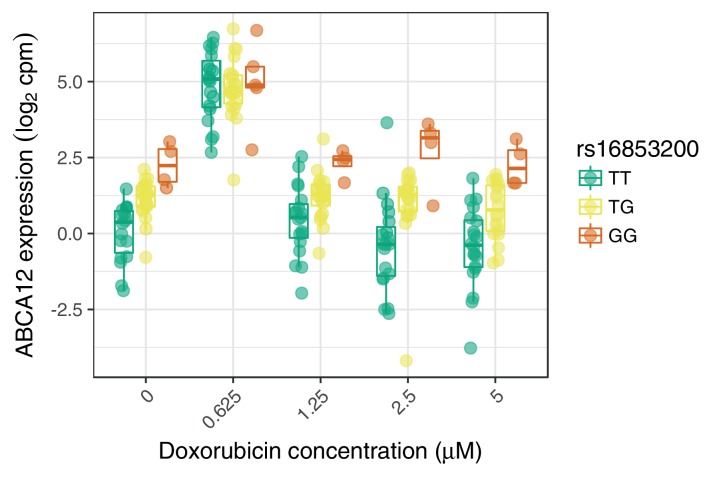
An example response expression QTL that may act through buffering at high expression levels.

**Figure 2—figure supplement 4.**
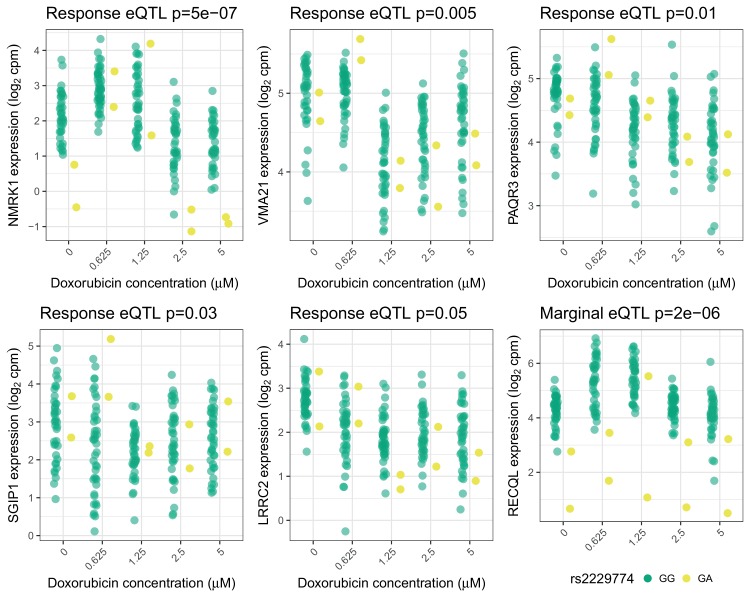
Trans-eQTLs for rs2229774 in *RARG*. Trans-eQTLs (5 response and one marginal) at 5% FDR for rs2229774, a non-synonymous coding variant in *RARG* which is associated with ACT (Aminkeng et al., 2015). Only two individuals are heterozygous. p-values shown have been adjusted for multiple testing by Benjamini Hochberg.

To characterize the detected reQTLs we assigned the response of the major and minor allele to one of the six clusters previously learned (Figure 1c), with heterozygotes expected to display the average of the two homozygous responses. 172 (46%) of reQTLs result in a qualitatively distinct response as determined by the two alleles being assigned to different clusters. The most common transition, occurring for 33 reQTLs, is that the major allele is associated with simple down-regulation (cluster 1) in response to doxorubicin, whereas the minor allele shows up-regulation at low concentration followed by down-regulation at higher concentration (cluster 2).

We further broke-down the significant reQTLs by considering the effect of genotype on expression at each concentration ($\beta_{c}$ in Equation 6). We normalized the effect sizes relative to the $\beta_{c}$ with the largest absolute value, i.e. we consider $\beta_{c}/\beta_{argmax|\beta_{c^{\prime }}|}$, so that the largest genotype effect always corresponds to a normalized value of 1. The resulting normalized effect profiles were split into nine clusters using k-means clustering (Figure 2d). The largest cluster (cluster 1, 85 reQTLs) represents reQTLs with a modest effect size at low concentrations (0,0.625μM) which is amplified at higher concentrations (Figure 2e shows a highly significant example). Cluster two corresponds to reQTLs whose effect size is attenuated at the 0.625μM treatment: examples of reQTLs in this cluster tend to be associated with higher expression level at the 0.625μM treatment (e.g. rs16853200’s association with ABCA12 response, Figure 2—figure supplement 3).

A non-synonymous coding variant in *RARG*, rs2229774, was previously associated with ACT (Aminkeng et al., 2015). Since *RARG* codes for a transcription factor we searched transcriptome-wide for rs2229774 trans-eQTLs: genes where the expression response to doxorubicin appears to be different for different *RARG* alleles. Only two of the individuals in our panel carry the alternative A allele (as heterozygotes) with the rest being homozygous reference (GG). While this limits statistical power, suez detects one marginal effect (*RECQL*) and five response trans-eQTLs (*NMRK1*, *VMA21*, *PAQR3*, *SGIP1* and *LRRC2*) at 5% FDR (Figure 2—figure supplement 4). Interestingly PAQR3, a membrane protein localized to the Golgi apparatus, is a negative regulator of antioxidant response through the Nrf2-Keap1 pathway (Zhang et al., 2016). *LRRC2* is a mitochondrial protein whose RNA expression level has been previous linked with heart failure (McDermott-Roe et al., 2017).

### Doxorubicin exposure reduces splicing fidelity

**Figure 3.**
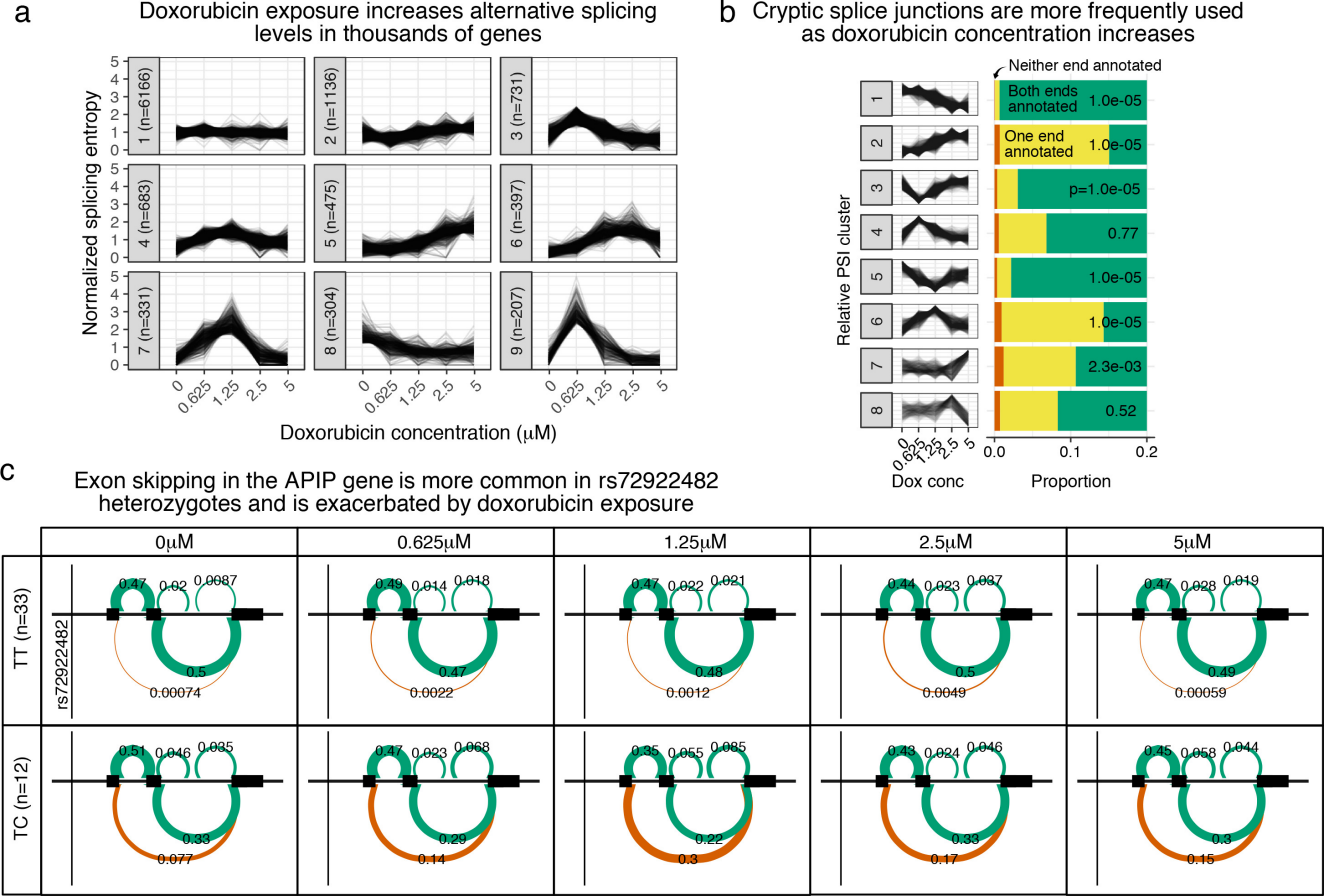
Doxorubicin exposure significantly impacts alternative splicing. (**a**) The entropy of splicing choices increases in response to doxorubicin exposure. We measured splicing entropy at different concentrations within LeafCutter ‘Alternative Splicing Clusters’ (ACSs) and clustered these into patterns of entropy change. (**b**) We separated introns differentially excised with ΔΨ>0.1 into eight clusters based on their relative excision level at each concentration. Introns in clusters corresponding to increased excision at higher doxorubicin concentrations (e.g. cluster 2) are far more likely to use a cryptic (unannotated) splice site at at least one end. p-values shown are for a hypergeometric test of that cluster against all others. (**c**) We mapped 42 ASCs with response splicing QTLs, variants that modulate the differential splicing response to doxorubicin.

Oxidative stress, a major downstream consequence of doxorubicin exposure, disrupts splicing of individual genes including *HPRT*, *POLB* (Disher and Skandalis, 2007), and *SMA* (Seo et al., 2016). We queried the extent to which doxorubicin exposure disrupts splicing patterns across the transcriptome using LeafCutter (Li et al., 2017). Across all samples LeafCutter detected 27769 alternative splicing ‘clusters’ (referred to here as ‘ASCs’ to avoid confusion with k-means clusters), which correspond approximately to splicing events, with a median of 3.0 splice junctions per ASC. Of 17755 ASCs with sufficient coverage to test, 10430 (59%), corresponding to 6398 unique genes, showed an effect of doxorubicin exposure on splicing outcomes (5% FDR, Supplementary files 7–8). To characterize these changes we calculated the entropy of the splicing choices made for each significant ASC at each concentration and used k-means clusters patterns of change in entropy (Figure 3a ). The largest cluster has 6166 ASCs (59%), and corresponds to the null of no clear change in entropy across concentrations. Clusters 2 (n=1136) and 5 (n=475) correspond to increasing entropy with concentration, and clusters 3, 4, 6, 8 and 9 correspond to the maximum entropy being at different concentrations and reaching different maximum levels. Interestingly, only the relatively small cluster 8 (n=304,3% of ACSs) corresponds to a reduction in entropy at higher concentrations, suggesting the dominant behavior is reduced splicing fidelity and increased alternative splicing in response to doxorubicin.

**Figure 3—figure supplement 1.**
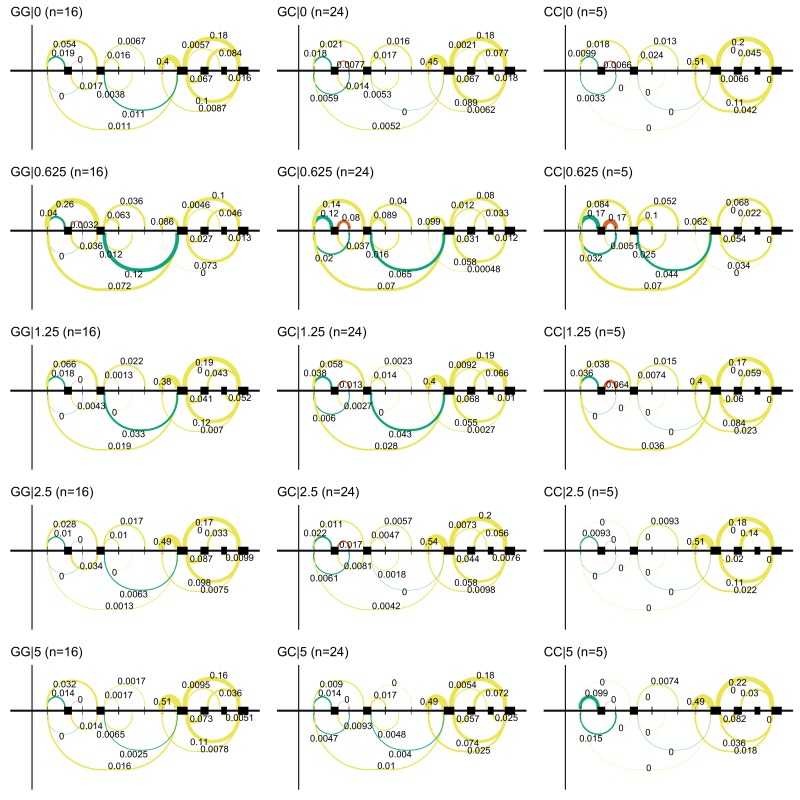
Alternative TSS use in NDUFAF6 on doxorubicin exposure. Doxorubicin exposure results in the use of a downstream alternative TSS for NDUFAF6 which uncovers an association between rs896853 genotype and inclusion of the exon at chr8:94975318–94975415.

**Figure 3—figure supplement 2.**
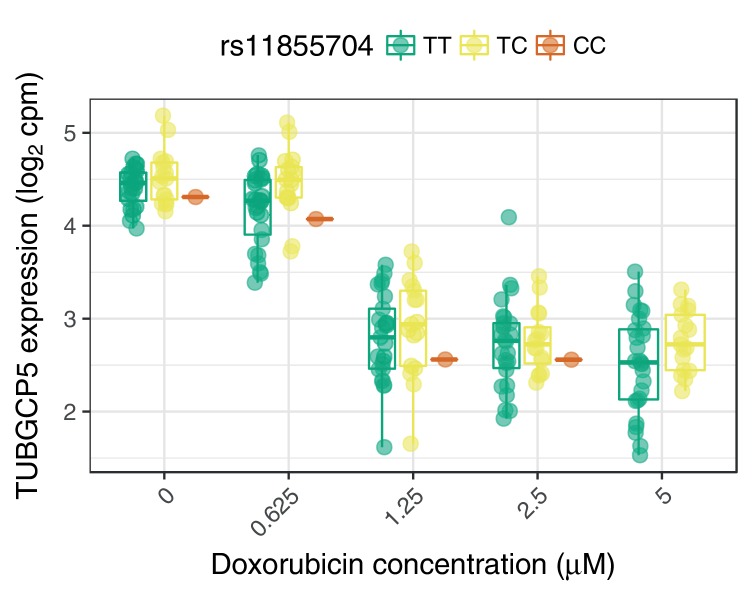
The ACT-sensitivity variant rs28714259 weakly modulates TUBGCP5’s response to doxorubicin. rs28714259, the one replicated variant from (Schneider et al., 2017) is in high LD ($R^{2}=0.98$) with rs11855704, which is a nominally significant (p=0.036) eQTL for TUBGCP5. TUBGCP5 is strongly down-regulated in the presence of doxorubicin.

We further tested the hypothesis that splicing fidelity decreases in the presence of doxorubicin by comparing patterns of intronic percent excised (Ψ) with canonical vs cryptic (unannotated) splice site usage. We clustered the 7792 introns in significantly differentially spliced ASC, that have a change in percent excised ΔΨ>0.1 for some pair of concentrations, into eight response patterns based on their relative excision proportions across concentrations. For each cluster we calculated the proportion of member introns with neither end annotated, one end unannotated, or both ends annotated (Figure 3b). The clusters representing increased Ψ with concentration (clusters 2, 4, 6 and 7) all show enrichment for cryptic splice site usage. The two most populous clusters (1 and 2) correspond to Ψ decreasing and increasingly continuously with doxorubicin concentration, respectively, and the difference in levels of cryptic splicing is extremely apparent (hypergeometric $p<2\times 10^{-16}$, odds ratio for one annotated end vs two is 28.0).

We additionally used LeafCutter quantification of percentage spliced in (PSI) for each splice junction to map splicing QTLs (sQTL) and response-splicing QTLs (rsQTL) using suez. We tested SNPs within 100 kb of either end of the splice junction. At 5% FDR we found 467 ASCs with a marginal effect sQTL (Supplementary file 9) and 42 with a rsQTL (Supplementary file 10). An example rsQTL is rs72922482’s association with inclusion of exon 2 of APAF1 Interacting Protein (APIP). Under the major T allele exon skipping is extremely rare: the LeafCutter PSI for the spanning junction ranges from 0.00059 to 0.0049 across concentrations (Figure 3c). In rs72922482 heterozygotes, however, the exon is skipped in a significant proportion of transcripts, and this effect is most pronounced in the data collected after treatment at 1.25μM, with approximately 50% exon inclusion, suggesting the minor C allele results in very low inclusion of the cassette exon. Another interesting example is *NDUFAF6*, another mitochrondrial Complex I protein, where doxorubicin exposure (particularly at 0.625μM) results in increased use of an alternative downstream transcription start site (TSS) which unmasks the influence of rs896853 on a cassette exon between the two alternative TSS (Figure 3—figure supplement 1).

### Transcriptional response to doxorubicin is predictive of in-vitro cardiac-damage indicator troponin

**Figure 4.**
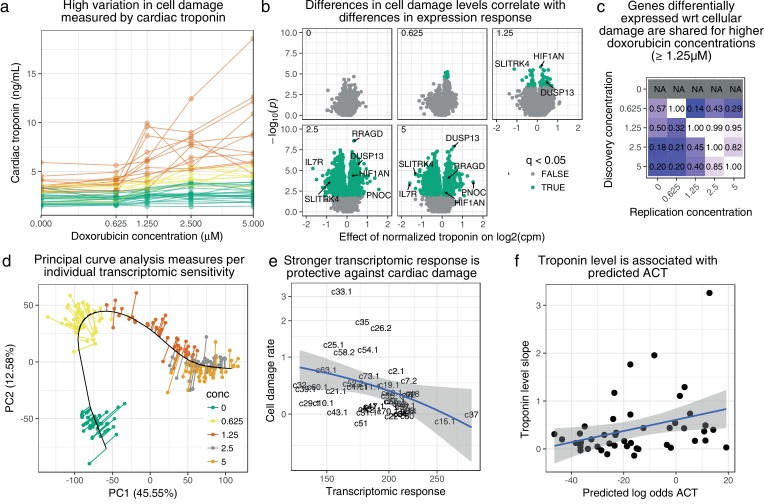
Transcriptomic response is predictive of doxorubicin induced damage as measured by cardiac troponin. (**a**) We measured media levels of cardiac troponin, a sensitive and specific test for myocardial cell damage, in response to doxorubicin, across all cell lines. (**b**) We performed differential expression analyses with respect to troponin at each concentration separately, and observed more differentially expressed genes at higher concentrations corresponding to an increased dynamic range of troponin levels. (**c**) We took differentially expressed genes (5% FDR) at each concentration and checked for ‘replication’ (nominal p<0.05)) at the other concentrations. Note that no differentially expressed genes were discovered in control condition (0μM). (**d**) We summarized gene expression response by first fitting a ‘principal curve’ following increasing doxorubicin concentration, and then measuring the rate of progression along this curve for each individual. (**e**) Increased transcriptomic response is associated with reduced cardiac troponin levels, suggesting that the bulk of expression changes we observe are in fact protective against cardiac damage. (**f**) We trained a model to predict ACT risk from gene expression response using available 3 v. 3 case/control data (Burridge et al., 2016) and applied this model to our data. Predicted ACT risk correlated significantly with the slope of troponin level (Spearman ρ=0.38,p=0.01), supporting the in vivo disease relevance of our IC system.

We used the level of cardiac troponin released into the culture media by lysed cardiomyocytes (see Methods, Supplementary file 11) to estimate damage occurring as a result of doxorubicin exposure at different concentrations. We observed significant variation in measurable damage caused by doxorubicin across individuals, with 13 of 45 cell lines having a significant correlation between doxorubicin dose and troponin measurement (Figure 4a). We first sought to determine whether the inter-individual variation in troponin in culture could be explained by variation in the overall gene expression response. Since we are interested in this case in inter-individual differences rather than differences between concentrations we normalized the troponin measurements to have 0 mean and variance of 1 across samples at each doxorubicin treatment. We found 96.1% (95% credible interval 91.5%−98.6%) or 91.5% of the variance in this normalized troponin level could be explained using gene expression levels (we excluded the troponin genes *TNNT1-3* and *TNNI1-3* from the analysis) at the corresponding doxorubicin concentrations, using a GREML-analysis (Yang et al., 2010) or leave-out-one cross validated (LOOCV) lasso (Tibshirani, 1996) respectively. The optimal lasso model included 118 genes (Supplementary file 12). To test whether gene expression mediates a link from genotype to troponin level we performed a transcriptome-wide association study (TWAS, Gamazon et al., 2015). For each gene we built an elastic-net predictor of expression at each doxorubicin concentration using SNPs within 100 kb, with 10-fold cross-validation to choose the regularization parameters. The fitted predictions (the ‘pre-validation’ values) represent the genetically-determined component of expression. We used the 3840 genes with a statistically significant genetic component (at 1% FDR) to predict troponin level using LOOCV lasso regression. 89% of the variance in normalized troponin level can be explained by the genetic component of 102 genes (Supplementary file 13). This analysis is analogous to two-stage least squares Mendelian randomization (Angrist and Imbens, 1995) analysis and therefore suggests the existence of a causal link from genotype through gene expression to troponin level, and highlights potential mediating genes. However, further assumptions — in particular that the SNPs and troponin level are independent conditional on gene expression — would be required to formally establish a causal connection.

To further explore the relationship between transcriptomic response and troponin presence in culture, we analyzed differential expression (DE) with respect to troponin measurement at each doxorubicin concentration separately. We found 0, 7, 78, 2984 and 2863 differentially expressed genes (5% FDR, Supplementary file 14) at the five concentrations respectively (Figure 4b). The most strongly DE gene (with respect to effect size) at the 5μM treatment is *DUSP13*, a known regulator of *ASK1*-mediated apoptosis (Park et al., 2010). The large number of DE genes at the 2.5μM and 5.0μM treatments are broadly shared (nominal replication rate 82 to 85%), and DE genes at the 1.25μM treatment generally represent the most strongly DE genes at the higher concentrations (Figure 4c).

To compare troponin measurements to transcriptomic response we determined an overall per-individual level of transcriptomic response with respect to doxorubicin concentration. To this end we fit a principal curve (Hastie and Stuetzle, 1989) through all gene expression samples, initializing the curve to pass sequentially through the successive doxorubicin concentrations (Figure 4d). Projecting every sample on the principal curve gives a single measure of ‘progression’ through response to doxorubicin at increasing concentrations. We then regressed these values against concentration for each individual to obtain a progression rate. We found the troponin measurement slope is significantly negatively correlated (Spearman ρ=−0.42,p=0.004, Figure 4e) with the transcriptomic response rate, suggesting that much of the gene expression program being activated in response to doxorubicin is in fact protective against cardiac damage.

Using previously published data (Burridge et al., 2016), we built a predictive model of ACT risk trained on RNA-seq of ICs exposed to 1μM doxorubicin from doxorubicin-treated patients who did (‘DOXTOX’, n=3) or did not (‘DOX’, n=3) develop ACT. Using lasso with fixed $\lambda =10^{-5}$ the optimal model included 17 genes as features (Supplementary file 15). We applied this model to our expression data from the 0.625μM treatment (since this concentration shows excellent concordance with the 1μM data of Burridge et al., see Figure 1—figure supplement 6) to obtain predicted log-odds of ACT. While these log-odds are unlikely to be well-calibrated due to differences in the training and test datasets, they may still accurately represent relative risk of ACT across our 45 individuals. Indeed, the log-odds correlated significantly with the troponin measurement slope (Spearman correlation p=0.01, Figure 4f), suggesting our troponin measurements, and by extension our expression response data, recapitulate in vivo cellular response to doxorubicin.

### Cardiomyocyte molecular QTLs show enrichment in ACT GWAS

**Figure 5.**
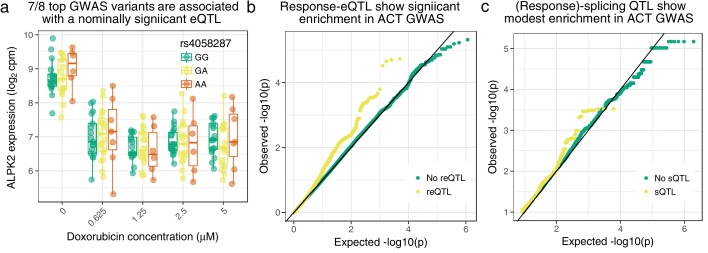
Cardiomyocyte molecular QTLs are enriched in meta-analyzed ACT GWAS (Schneider et al., 2017; Serie et al., 2017). (**a**) rs4058287 has a GWAS p-value of $9.68\times 10^{-6}$ and is a nominally significant eQTL (p=0.0016) for *ALPK2*, which is down-regulated in response to doxorubicin. (**b**) SNPs that have a response eQTL with $p<10^{-5}$ are enriched in GWAS variants with p<0.05 (hypergeometric test $p=2\times 10^{-37}$). (**c**) SNPs with a marginal or response splicing QTL at $p<10^{-5}$ show modest enrichment in GWAS p<0.005 (hypergeometric p=0.02).

To determine the disease-relevance of our molecular QTLs we obtained summary statistics for the largest ACT GWAS to date (Schneider et al., 2017). While this GWAS was not sufficiently powered to find genome-wide significant associations, 11 variants representing nine independent loci have $p<10^{-5}$, with the most significant (rs2184559) at $p=2.8\times 10^{-6}$. Of the 8 GWAS variants with $p<10^{-5}$ either tested in our eQTL mapping, or in high LD ($R^{2}>0.8$) with a tested SNP, seven have a nominally significant marginal eQTL (p<0.05, the 8th has p=0.07) and four have a reQTL with p<0.1. The one replicated variant in this GWAS, rs28714259, was not genotyped in our data but is in high LD ($R^{2}=0.98$) with rs11855704 which is a nominally significant marginal eQTL for tubulin gamma complex associated protein 5 (*TUBGCP5*, Figure 3—figure supplement 2). rs4058287 (GWAS p-value $9.68\times 10^{-6}$) has a marginal effect on Alpha-Protein Kinase 2 (*ALPK2*, also known as ‘Heart Alpha-Protein Kinase’ since it was discovered in mouse heart (Ryazanov et al., 1999) and is expressed in few other tissues (Melé et al., 2015)) expression (p=0.0016) as well as a weak interaction effect (p=0.06, see Figure 5a). Interestingly, *ALPK2* has been shown to upregulate DNA repair genes and to enable caspase-3 cleavage and apoptosis in a colorectal cancer model (Yoshida et al., 2012). The replicating variant from Aminkeng et al., 2015, rs2229774 only occurs in two individuals in our cohort (who are heterozygous) making eQTL mapping infeasible. Additionally we find a marginal effect eQTL (p=0.0017, Figure 5—figure supplement 1) on SLC28A3 for rs885004, which has previously been associated with ACT in a candidate gene study (Visscher et al., 2013). rs885004 is intronic, is in LD ($R^{2}=0.98$) with another ACT implicated variant, rs7853758 (Visscher et al., 2012), and falls in a DNase I hypersensitivity and H3K27ac peak present in numerous ENCODE cell lines (and is open in our ICs according to ATAC-seq data, see Figure 5—figure supplement 2).

**Figure 5—figure supplement 1.**
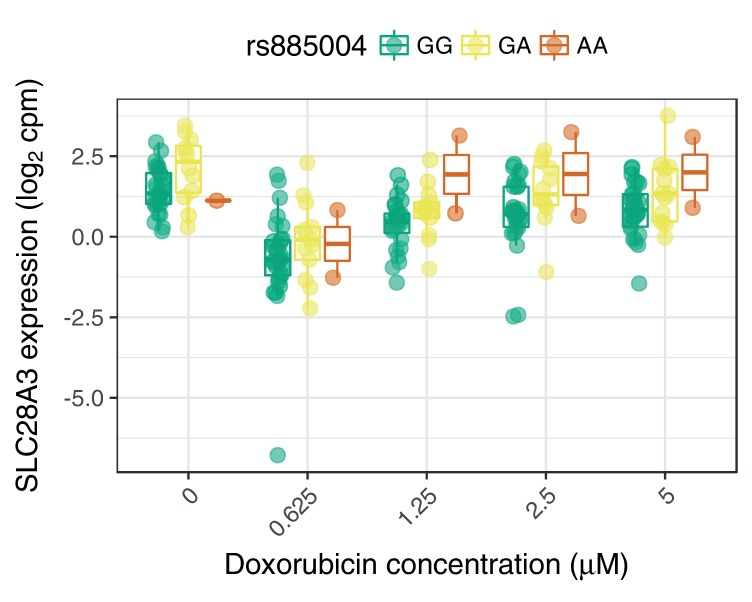
SLC28A3 expression may mediate the association of rs885004 with ACT-sensitivity. The intronic (to SLC28A3) variant rs885004, and the closely linked ($R^{2}=0.98$) synonymous variant rs7853758, have been associated with ACT by candidate gene studies (Visscher et al., 2012; Visscher et al., 2013). We find rs885004, but not rs7853758, has a significant marginal effect on SLC28A3 expression.

**Figure 5—figure supplement 2.**
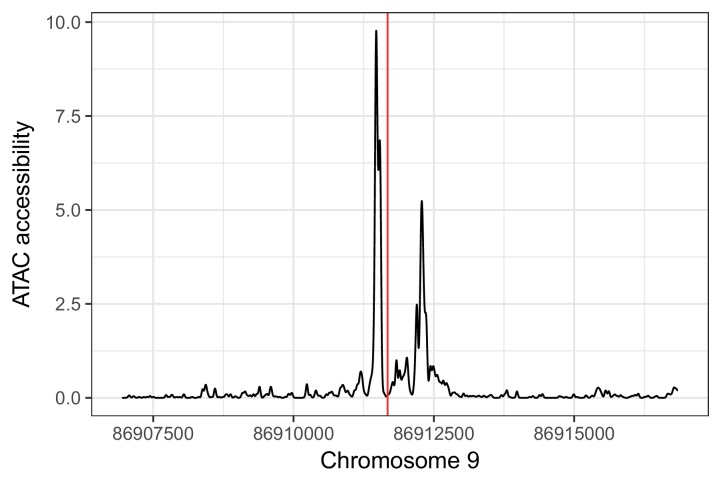
rs885004 falls in an IC ATAC-seq peak. rs885004 (shown in red) falls in an ATAC-seq footprint in (unrelated) iPSC-derived cardiomyocytes (unpublished data).

**Figure 5—figure supplement 3.**
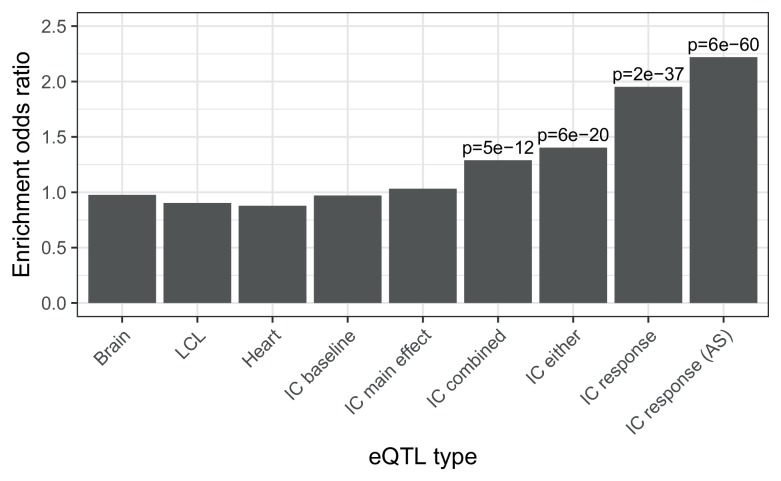
Enrichment of different classes of expression QTLs in ACT GWAS. Enrichment of low (<0.05) ACT GWAS p-values in different classes of expression QTLs ($p<10^{-5}$). First three are GTEx tissues (steady-state). **IC baseline:** iPSC-derived cardiomyocytes at baseline (no doxorubicin). **IC main effect:** marginal effect of genotype across concentrations. **IC combined:** jointly testing for a main effect or interaction effect with concentration. **IC either:** either a marginal or response eQTL. **IC response:** testing for an interaction effect between genotype and concentration. **IC response (AS):** as for IC response but using both total and allele-specific expression to map reQTLs.

**Figure 5—figure supplement 4.**
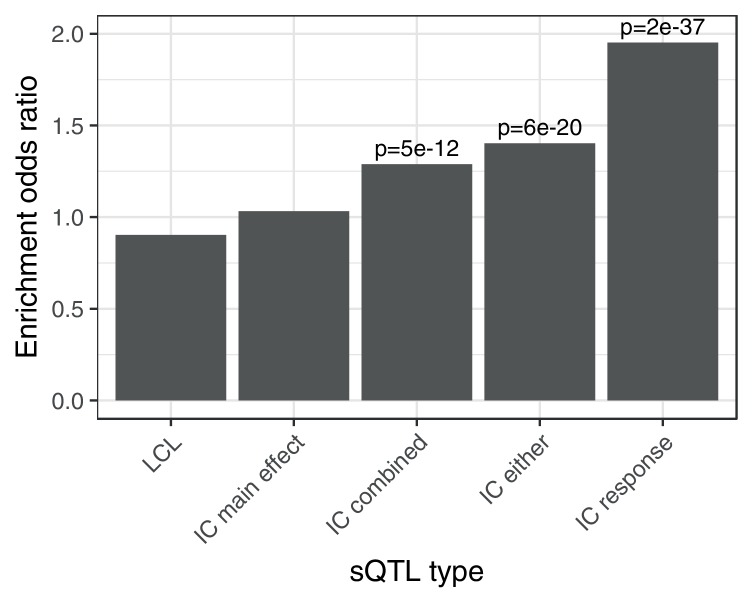
Enrichment of different classes of splicing QTLs in ACT GWAS. Enrichment of low (<0.05) ACT GWAS p-values in different classes of splicing QTLs ($p<10^{-5}$). **LCL:** LeafCutter splicing QTLs mapped in the Yoruban LCLs (Li et al., 2016). **IC main effect:** marginal effect of genotype across concentrations. **IC combined:** jointly testing for a main effect or interaction effect with concentration. **IC either:** either a marginal or response eQTL. **IC response:** testing for an interaction effect between genotype and concentration.

**Figure 5—figure supplement 5.**
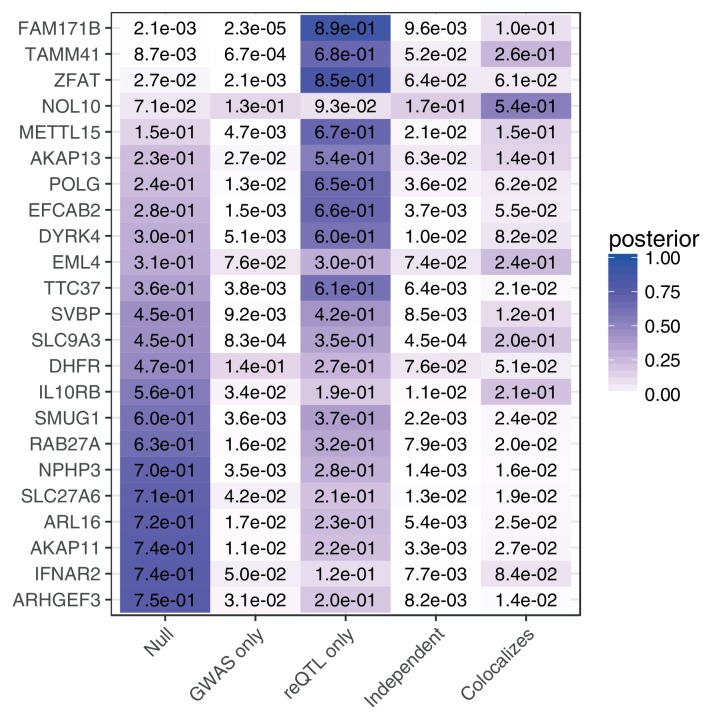
Colocalization analysis. Colocalization analysis for genes where the posterior probability of no association estimated by coloc is <0.75.

To determine whether our molecular QTLs are more useful than published QTLs for interpreting ACT risk variants we first sought to obtain the best powered GWAS data possible. Since the Schneider et al. GWAS was overall underpowered, we obtained additional ACT GWAS summary statistics from a more recent study (Serie et al., 2017) and performed a meta-analysis with Schneider et al. We used this data to assess whether there was detectable enrichment of low GWAS *p*-values for our regulatory QTLs. When considering eQTL with nominal p*<*10^−5^ (corresponding approximately to 5% FDR) we found no enrichment for GWAS p*<*0.05 for three GTEx tissues (heart, brain and lymphoblastoid cell lines—LCLs), our marginal effect eQTLs or baseline (no doxorubicin) only eQTLs (Figure 5—figure supplement 3). However, considering SNPs that are either main effect or response eQTL we see significant enrichment (one-sided hypergeometric p=6 × 10^−20^, OR = 1.40). Similarly for ‘combined’ eQTL where we explicitly test for any effect of genotype (main or interaction effect, see Methods) we see enrichment (p=5 × 10^−12^, OR = 1.29). Furthermore, focusing on response eQTL we see a stronger enrichment (p=2 × 10^−37^, OR = 1.95, Figure 5b), suggesting that the enrichment in combined eQTL is driven by this signal. Response eQTLs mapped using allelic-specific expression as well as total expression show the strongest enrichment (p=6 × 10^−60^, OR = 2.22). When considering splicing QTLs (Figure 5—figure supplement 4) we found no enrichment for marginal sQTLs mapped in LCLs (Li et al., 2016). Interestingly, in contrast to the total expression QTLs we found a significant enrichment (p=4 × 10^−17^, OR = 1.36, Figure 5c) for IC marginal sQTLs although the enrichment in response sQTLs was still higher in absolute terms (p=2 × 10^−5^, OR = 1.57). These findings are indicative that molecular response QTL mapping has potential for understanding the molecular basis of environmentally-dependent human disease.

Finally, we attempted colocalization analysis for our response eQTLs and meta-analyzed GWAS using *coloc* (Giambartolomei et al., 2014), a Bayesian method based on summary statistics. For each region (gene in our case) *coloc* infers a posterior probability for each of five possibilities: (H0) no association, (H1) association for the eQTL only, (H2) association for the GWAS only, (H3) independent variants, or (H4) colocalization to one variant. Out of 43 genes with a SNP with a reQTL at p*<*10^−5^ and GWAS SNP at p*<*0.05 *coloc* gave maximum posterior probability to the null hypothesis (H0, no association) for 32 genes, association only for reQTL (H1) for 9, and colocalization (H4) for one gene, *NOL10* (posterior probability of colocalization 0:54, Figure 5—figure supplement 5, Supplementary file 16). While these results suggest our data is not sufficiently well-powered for colocalization analysis, we note that the posterior probability of colocalization (H4) is higher than that for independent signal (H3) in 40/43 tested genes.

## Discussion

Human iPSC-derived somatic cells provide a powerful, renewable and reproducible tool for modeling cellular responses to external perturbation in vitro, especially for non-blood cell-types such as cardiomyocytes which are extremely challenging to collect and even then are typically only available post-mortem. We established a sufficiently large iPSC panel to effectively query the transcriptomic response of differentiated cardiomyocytes to doxorubicin. We were also able to characterize the role of genetic variation in modulating this response, both in terms of total expression and alternative splicing. There are, of course, caveats associated with using an in vitro system, which may not accurately represent certain aspects cardiac response to doxorubicin in vivo. That said, the replication of GTEx heart eQTLs, association of troponin levels with predicted ACT-risk (Burridge et al., 2016), and the observed GWAS enrichment, all support the notion that the IC system recapitulates substantial elements of in vivo biology. It is challenging to quantify this agreement, and there are in vivo factors that are certainly not represented. For example, excessive fibrosis may contribute to ACT (Cascales et al., 2013; Zhan et al., 2016; Farhad et al., 2016; Heck et al., 2017), although is unclear how substantial this contribution is as well as whether fibroblasts are directly activated by doxorubicin exposure or simply respond indirectly to cardiomyocyte damage. While our FACS analysis shows cardiomyocytes are the dominant cell type in our cultures, heterogeneity remains and other cell types could be mediating some of the observed changes.

For many diseases such as ACT which involve an environmental perturbation it is reasonable to suppose that eQTLs detected at steady-state are only tangentially relevant when attempting to interpret disease variants. Such concerns motivated us to focus on response eQTLs, that is, variants that that have functional consequences under specific cellular conditions because they interact, directly or indirectly, with the treatment. We used a statistical definition of reQTLs corresponding to cases where gene expression levels are significantly better explained using a model including an interaction term between genotype and treatment (represented as a categorical variable), compared to a model with only additive effects for genotype and treatment. Our characterization of the detected reQTL demonstrates that these variants are indeed candidate drivers of differences in individual transcriptomic response to doxorubicin. The strongest reQTL effects correspond to completely different response patterns for the major and minor alleles, while weaker effects correspond to more subtle modulation of the same response pattern. We note that it is not necessarily the case that such reQTLs are the only functionally relevant eQTLs. eSNPs with a marginal (additive) effect on expression of a gene responsive to doxorubicin (as most genes are) could still be important if the relationship between expression and ACT-risk is nonlinear, for example involving thresholding effects.

We observed a statistical enrichment of expression and (to a lesser extent) splicing QTLs in ACT GWAS. However, with no reproducible genome-wide significant associations available, fine-mapping of causal variants remains fraught. We anticipate our findings will be increasingly valuable as larger-scale ACT GWAS become available.

We derived ICs from healthy individuals so we do not known which individuals would develop ACT if they required anthracycline treatment. Mapping molecular response QTLs in larger panels of ICs from patients treated with anthracyclines who do or do not develop ACT symptoms would allow stronger conclusions to be drawn about the contribution of the detected (r)eQTLs to disease etiology.

We used a panel of Hutterites individual since this homogeneous population offers unique advantages for mapping genetic traits: exposure to a fairly uniform environment and less variable genetic background, despite still representing much of European diversity (Newman et al., 2004). However, the genetic basis of ACT susceptibility is likely complex and some relevant genetic variation may not be well represented in this cohort.

Finally, an interesting observation in our study is that splicing fidelity is reduced upon doxorubicin exposure. This is not completely unexpected since a key downstream side-effect of doxorubicin is increased oxidative stress, which has been previously associated with dysregulated splicing of specific genes (Disher and Skandalis, 2007; Seo et al., 2016). Our finding that this effect is prevalent across the transcriptome poses further questions about what known effects of doxorubicin might, in fact, be mediated by changes in RNA splicing.

## Materials and methods

### Sample collection and genotyping

Generation of lymphoblastoid cell lines (LCLs) and genome-wide genotyping of many individuals from a multi-generational pedigree were performed previously. Briefly, lymphocytes were isolated from whole blood samples using Ficoll-Paque and immortalized using Epstein Barr Virus (EBV) (Cusanovich et al., 2012; Cusanovich et al., 2016). Phased genotypes were obtained by combining pedigree information, genotypes from SNP arrays, and genotypes from whole genome sequencing of related individuals (Livne et al., 2015).

### iPSC reprogramming

We reprogrammed 75 LCLs to iPSCs using episomal plasmid vectors, containing *OCT3/4*, *p53* shRNA, *SOX2*, *KLF4*, *L-MYC*, and *LIN28* which avoids integrating additional transgenes (Okita et al., 2011). Initially, the lines were generated on mouse embryonic fibroblasts (MEF), which coated the well and served as feeder cells to create an environment supportive of pluripotent stem cells. The colony was then mechanically passaged on MEF and tested for expression of pluripotency-associated markers by immunofluorescence staining and RT-PCR. The lines were passaged for at least 10 weeks on MEF to ensure lines had stabilized.

All iPSC lines were characterized as described previously (Gallego Romero et al., 2015). Briefly, we initially performed qPCR using 1μg of total RNA, converted to cDNA, from all samples to confirm the endogenous expression of pluripotency genes: *OCT3/4*, *NANOG*, and *SOX2* (Figure 1—figure supplement 1). We next confirmed pluripotency using PluriTest (Müller et al., 2011). All samples were classified as pluripotent and had a low novelty score (Figure 1—figure supplement 2). Additionally, we confirmed the ability of all iPSC lines to differentiate into the three main germ layers using the embryoid body (EB) assay (Supplementary Data). Finally, we tested for the presence and expression of the EBV gene EBNA-1 using PCR (Figure 1—figure supplement 3, Figure 1—figure supplement 4). We tested all samples for both genomic integrations and vector-based EBV. If the cells were positive (two positive and one indeterminate case was identified), we further tested the origin of the EBV (genomic or episomal) using primers specific to the LMP-2A gene found in EBV or part of the sequence specific to the episomal plasmid (Figure 1—figure supplement 3). We concluded that two lines still had EBV present in the genome, this was also reflected in EBNA-1 gene expression for these individuals (Figure 1—figure supplement 4). We retained these individuals because they passed all quality control metrics and were not outliers based on genome-wide gene expression. It should also be noted that gene expression levels are extremely similar between iPSC lines. This relative homogeneity further demonstrates the quality of our iPSC lines. In summary, all iPSC lines showed expression of pluripotent genes quanti1ed by qPCR, generated EBs for all three germ layers, and were classified as pluripotent based on PluriTest.

### Cardiomyocyte differentiation

iPSC lines were transitioned to feeder-free conditions, which was necessary to prime the iPSCs for differentiation. Next we differentiated the iPSCs to cardiomyocytes (Lian et al., 2013; Burridge et al., 2014). iPSC lines were covered with a 1:60 dilution matrigel overlay for 24 hr. On day 0 iPSC lines were treated with 12μM of the *GSK3* inhibitor, CHIR99021, in RPMI+ B27 medium (RPMI1640, 2 nM L-glutamine and 1x B27 supplement minus insulin) for 24 hr at which time media was replaced with fresh RPMI + B27. 72 hr after the addition of CHIR99021 (Day 3), 2μM of the Wnt inhibitor Wnt C-59 was added for 48 hr. Fresh RPMI + B27 was added on Days 5, 7 and 10. Beating cells appeared between Days 8–10. These cardiomyocytes consisted of ventricular, atrial and pacemaker-like cells. The cells formed thick layers and contract throughout the well. Metabolic selection was used to purify the cardiomyocytes (Tohyama et al., 2013) from Day 14 to Day 20 when glucose-free RPMI media supplemented with the components essential for cardiomyocyte differentiation (Burridge et al., 2014), ascorbic acid and human serum albumin, together with lactate, a substrate uniquely metabolized by cardiomyocytes, was added to cells. Because this lactate media can only be metabolized by cardiomyocytes, the non-cardiomyocytes in the culture were removed over the 6 day treatment. On day 20 the cardiomyocytes, now at a high cTnT purity, were replated for experiments in media that contains only galactose and fatty acids as an energy source. This galactose media forces the cardiomyocytes to undergo aerobic respiration, rather than anaerobic glycolysis common in cultured cells.

### IC purity analysis

At day 20 when ICs were plated for doxorubicin exposure, a portion of cells were collected to assess purity. Cells were harvested from plates by incubating with TrypLE Express (Thermo Fisher Scientific, Cat. No 12604013) for 5 min at 37°C. Once removed, cells were manually dissociated further by passing through a 100μm and then 40μm strainer to create a single cell suspension. Cells were then resuspended, fixed, and permeabilized (*Foxp3*/Transcription Factor Staining Buffer Set; eBioscience/Affymetrix, Cat. No 00-5523-00) according to the manufacturer’s instructions. Cells were stained with directly conjugated antibodies to cTnI (Alexa Fluor647 Mouse Anti-Cardiac Troponin I Clone C5; BD Biosciences, Cat. No 564409) and cTnT (PE Mouse Anti-Cardiac Troponin T Clone 13–11; BD Biosciences, Cat. No 564767). The Zombie Violet^TM^ Fixable Viability Kit (BioLegend, Cat. No 423113) was used to assess cell viability at the time of fixation. The following isotype controls were used: Alexa Fluor647 Mouse IgG2b, κ Isotype Control Clone 27–35 (BD Biosciences Catalog No. 558713) and PE Mouse IgG1, κ Isotype Control Clone MOPC-21 (BD Biosciences Catalog No. 554680). Cells were analyzed using a FACS Canto or LSR-II flow cytometers (BD Biosciences), and the data were analyzed with FlowJo software (v10.0.7, Tree Star). All gates were established such that <2% of cells stained with isotype controls were positive and dead cells were excluded.

### Doxorubicin exposure

We incubated the cardiomyocytes in 0, 0.625, 1.25, 2.5, or 5 μM doxorubicin. After 24 hr, we collected the serum and cells from each condition. From the serum, we measured cardiac Troponin T levels using the ABNOVA Troponin I (Human) ELISA kit (cat. no. KA0233). From the cells, we extracted RNA for sequencing. Cells from each individual were treated separately, but batches of experiments were performed on different days. Each treatment batch contained 1 to 4 individuals. RNA quality was assessed with the Agilent Bioanalyzer.

### RNA-sequencing

We prepared libraries using the Illumina TruSeq Library Kit and generated 50 bp single-end reads on a HiSeq 4000 at the University of Chicago Functional Genomics Facility. We confirmed sequencing quality using FastQC and MultiQC (Ewels et al., 2016). We confirmed sample identity by (1) comparing allelic counts (quantified using samtools mpileup [Li et al., 2009]) of exonic SNPs to the known genotypes and (2) running verifyBamID (Jun et al., 2012).

### Expression quantification

We aligned RNA-seq reads using STAR version 2.5.2a (Dobin et al., 2013) to GRCh38/GENCODE release 24. We counted reads using feature Counts (Liao et al., 2014) and calculated counts per million reads (cpm) using `cpm` from the `edgeR` ‘R package (version 3.18.1) (Robinson et al., 2010). We discarded samples with $<10^{7}$ exonic reads and genes with median $\log_{2}\left(cpm\right)$ less than 00.

### Differential expression analysis

We performed differential expression (DE) analysis across all five doxorubicin concentrations jointly, using either a linear model on quantile normalized cpm value or Spearman correlation, followed by Benjamini-Hochberg False Discovery Rate (FDR) control. Since the vast majority of genes showed differential expression we did not investigate better powered DE methods such as DESeq2.

We clustered genes into ‘response patterns’ using a K-component mixture model

(1) $$\begin{array}{rl} \pi & ∼\text{Dir}\left(1/K,\cdots ,1/K\right)\\ z_{g}|\pi & ∼\text{Discrete}\left(\pi \right)\\ y_{ngc}|z_{g}=k,\theta & ∼N\left(\theta_{ck},\sigma^{2}\right) \end{array}$$

where π is a prior probability vector over cluster assignments, Dir is the Dirichlet distribution, $z_{g}$ is cluster from which gene g is generated, $y_{ngc}$ is the expression of gene g in individual n at concentration c, $\theta_{ck}$ is the mixture parameter (mean) across concentrations for cluster k, and $\sigma^{2}$ is a shared noise variance. We marginalize (sum) over $z_{g}$ and optimize with respect to π,θ,σ using the rstan R package (Carpenter et al., 2016) (version 2.16.2). The hyperparameters of the Dirichlet distribution are set such that in the limit of large K the model approximates a Dirichlet process mixture (MacEachern and Müller, 1998) which automatically learns of an appropriate number of mixture components to use from data.

Gene set and promoter motif enrichment were performed using HOMER v4.9.1 (Heinz et al., 2010) using default parameters and without de novo motif search.

### Response eQTL mapping

We developed an extension of the PANAMA (Fusi et al., 2012) linear mixed model (LMM) framework to map eQTLs and response eQTLs while accounting for latent confounding, which we call suez. suez entails a two step procedure. Step one is used to learn latent factors from all genes, using the model

$$\begin{array}{rl} y_{ncg} & =\sum_{k}W_{kg}x_{nck}+u_{ng}+v_{cg}+\xi_{ncg}+\epsilon_{ncg}\\ W_{kg} & ∼N(0,\sigma_{k}^{2})\;factor\;loadings/coefficients\\ u_{ng} & ∼N(0,\sigma_{u}^{2})\;individual\;random\;effects\\ \xi & ∼MVN(0,\sigma_{\xi }^{2}\Sigma )\;kinship\;random\;effect\\ \epsilon & ∼MVN(0,\text{diag}(\sigma_{\epsilon }^{2}))\;noise \end{array}$$

where $x_{nck}$ are latent factors, $v_{cg}$ are per gene, per concentration fixed effects. We integrate over W,u,ξ and ϵ, which results in a per gene multivariate normal,

(2) $$y_{:g}∼MVN\left(Vv_{:g},\sum_{k}\;\sigma_{k}^{2}x_{:k}x_{k:}^{T}+\sigma_{u}^{2}U+\sigma_{\xi }^{2}\Sigma +\sigma_{e}^{2}I\right),$$

where $y_{:g}$ refers to the vector of expression for gene g across all individuals and concentrations (i.e. all ‘samples’ where a sample is an individual-concentration pair), V is a matrix mapping concentrations to samples (i.e. $V_{sc}=1$ iff sample s is at concentration c) and U is a matrix of which samples are for the same individual (i.e. $U_{ss^{\prime }}=1$ if sample s and sample $s^{\prime }$ come from the same individual). We optimize x,v and the variances $\left\{\sigma_{u}^{2},\sigma_{k}^{2},\sigma_{\xi }^{2},\sigma_{\epsilon }^{2}\right\}$ jointly across all genes g.

In step 2 we test individual gene-SNP pairs while accounting for confounding using the covariance matrix

(3) $$\Sigma_{\pi }=\sum_{k}\;\sigma_{k}^{2}x_{:k}x_{k:}^{T}+\sigma_{u}^{2}U+\sigma_{\xi }^{2}\Sigma$$

which includes both latent confounding, individual random effects and similarity due to kinship. We consider three LMMs, all with the same parameterization of the covariance $\sigma_{\pi }^{2}\Sigma_{\pi }+\sigma_{e}^{2}I$ where $\sigma_{\pi }^{2}$ and $\sigma_{e}^{2}$ are optimized along with the fixed effects to allow the extent to which each gene follows the global covariance pattern to be adapted. The simple structure of this covariance also allows pre-computation of the eigen-decomposition of $\Sigma_{\pi }$ which enables linear (rather than cubic) time evaluation of the likelihood and its gradient.

Model 0 involves no effect of the SNP (and can therefore be fit once for a gene) and a fixed effect for concentration. Model 1 adds a marginal effect of the SNP genotype dosage d. Finally model 2 adds an interaction effect between concentration and genotype, which is equivalent to a concentration-specific genotype effect. In summary:

(4) $$\text{Model }0:\;E[y_{ncg}]=v_{cg}$$

(5) $$\text{Model }1:\;E[y_{ncg}]=v_{cg}+\beta d_{n}$$

(6) $$\text{Model }2:\;E[y_{ncg}]=v_{cg}+\beta_{c}d_{n}$$

We optimize $\sigma_{\pi }^{2}$, $\sigma_{e}^{2}$ and the regression coefficients for each of the three models separately, and use likelihood ratio tests (LRT) to compare the models. Comparing Model 1 vs 0 (one degree of freedom) tests whether there is a marginal effect of the variant. Comparing Model 2 vs 1 (C−1=4 degrees of freedom, where C is the number of conditions/concentrations) tests whether there is an interaction effect, i.e. whether the genetic effect on expression is different at different concentrations (or equivalently whether the response to doxorubicin is different for different genotypes). Finally Model 2 vs 0 (C=5 degrees of freedom) tests whether there is any effect of genotype on expression, either in terms of a marginal or concentration-specific effect (we refer to these as ‘combined’ eQTL). We use the conservative approach of using Bonferroni correction across SNPs for a gene, followed by Benjamini-Hochberg FDR control.

We quantile normalize the expression levels across all samples for each gene to a standard normal distribution so that the distributional assumptions of our linear mixed model are reasonable. However, optimizing the variance parameters $\sigma_{\pi }^{2}$ and $\sigma_{e}^{2}$ means that the $\chi^{2}$ distribution for the LRT will only hold asymptotically and p-values for finite sample sizes will tend to be somewhat anti-conservative. To account for this for response-eQTLs, we use a parametric bootstrap since there is no fully valid permutation strategy for testing interaction effects. This involves first fitting Model 1 and then simulating new expression data under the fitted model. Models 1 and 2 are then (re)fit to this data and compared using an LRT. We then perform Bonferroni correction across SNPs for each gene to obtain an empirical null distribution of per gene p-values which we use to estimate the true FDR for our response-eQTL results.

For significant reQTLs we assigned the response of the minor allele and major allele to the previously determined clusters using the model

ync|zA,za,θ∼N(12dnθczA+12(2−dn)θcza,σ2),

where $y_{nc}$ is the expression for individual n at concentration c, $z_{A}$ and $z_{a}$ are the cluster assignments for the major and minor allele respectively, $d_{n}\in \{0,1,2\}$ is the genotype dosage, and θ and $\sigma^{2}$ are fixed at the values learned in Equation 1. For each reQTL separately we calculate the likelihood of y given all possible pairs of assignments $\left(z_{A},z_{a}\right)$ and choose the maximum likelihood solution.

As for all k-means clustering in the paper, we used KMeans_rcpp function of the R package ClusterR v1.0.6, taking the best of 10 initializations using the k-means++ option, to cluster the normalized genotype effect profiles of the significant associations. The choice of 9 clusters was determined manually.

### Using allelic expression

We (Knowles et al., 2017; van de Geijn et al., 2015) and others (Kumasaka et al., 2016) have demonstrated that modeling allele-specific expression can improve power to detect both *cis* eQTLs (van de Geijn et al., 2015; Kumasaka et al., 2016) and reQTLs (Knowles et al., 2017). Here we employ a combination of ideas from these methods:

- We assume our computational phasing between the regulatory and exonic SNP(s) is correct, since we have previously shown that errors in phasing reduce power but do not inflate false positives (van de Geijn et al., 2015).
- We use a beta-binomial (denoted BB) likelihood allowing exact likelihood calculations and straightforward maximum likelihood parameter estimation via LBFGS in Stan (Carpenter et al., 2016). We use the parametrization BB(n,p,γ) where n is the total count, p is the mean, and c is the concentration. The usual pseudo-count parametrization is recovered as a=pc,b=(1−p)c.
- We model multiple exonic SNPs per gene to ease integration with the total expression signal from suez.

Under the hypothesis that the allelic effect of the test regulatory SNP varies across concentrations (analogous to Model 2 in Equation 6), we have

(7) $$y_{nkc}|r_{nkc},\phi_{nk}∼BB(r_{nkc},\sigma (\mu +\phi_{nk}\beta_{c}),\gamma )$$

where there are K exonic SNPs in a gene, with alternative allele counts $y_{nkc}$ and read coverage $r_{nkc}$. σ is the logistic function. μ is an intercept term to account for reference mapping bias, and γ is a per-gene concentration (reciprocal of over dispersion) parameter. We regularize γ using a Γ(1.001,0.001) prior. $\phi_{nk}\in \{-1,0,+1\}$ is the phased heterozygosity of the test regulatory SNP in individual n, with $\phi_{nk}=0$ if the regulatory SNP is homozygous (these individuals are included to help estimate μ and c) and $\phi_{nk}=1$ or −1 if the regulatory SNP is heterozygous and in phase (1) or opposite phase (-1) with SNP k. $\beta_{c}$ is the effect size in concentration c. The null (no interaction effect, corresponding to Model 1 in Equation 6) is that $\beta_{c}=\beta$ for all all c.

To integrate evidence from total and allelic specific expression it is valid to add the log likelihood ratios, which can be seen as either fitting one model with likelihood terms for the two components, or as a result of $\chi^{2}$ random variables being closed under addition. This approach has substantially better power than applying Fisher’s combined probability test to p-values from testing the total and allelic expression components separately. Twice the summed log likelihood ratio is asymptotically $\chi^{2}$ with degrees of freedom being simply the sum of the degrees of freedom of the two components (so usually 4+4=8 in our case). In practice we only fit the total expression model if there are at least 5 alternative alleles observed for the test regulatory SNP, and only fit the allele-specific model if there are at least 2000 supporting allelic reads for the gene, so some regulatory SNPs are only tested using one component or the other. In addition, for some genes whose expression is very low for specific concentrations there may be no allelic reads for a concentration, in which case the degrees of freedom for the allele-specific component will be reduced since no $\beta_{c}$ is learned for that concentration.

### Assessing agreement with GTEx eQTLs

We initially compared our eQTLs to GTEx eQTLs by estimating Storey’s π_1_ (Storey and Tibshirani, 2003), using the q value R package v2.8.0, for GTEx nominal *p*-values for our significant eQTLs (at a nominal p*<*10^−5^). While the GTEx heart tissues show higher replication than most tissues, surprising tissues ranked higher (Figure 2—figure supplement 1). We reasoned that differential power across the GTEx tissues due to differing sample size and noise levels confound this simple approach. We therefore used an extension of Storey’s π_1_ to test for overlap between two sets of *p*-values. For each GTEx tissue we fit (using LBFGS in Stan) a mixture model

(8) $$\left(p_{i1},p_{i2})∼\pi_{00}U(p_{i1})U(p_{i2})+\pi_{10}B(p_{i1}|a_{1},b_{1})U(p_{i2})+\pi_{01}U(p_{i1})B(p_{i2}|a_{2},b_{2})+\pi_{11}B(p_{i1}|a_{1},b_{1})B(p_{i2}|a_{2},b_{2}\right)$$

where $p_{ij}$ is the p-value for SNP-gene pair i in tissue j, U is the uniform distribution on [0,1] (corresponding to p-values coming from the null) and B is the beta distribution (corresponding to non-null p-values).$\pi_{00},\pi_{01},\pi_{10},\pi_{11}$ correspond to mixture weights are estimates of the proportion of SNP-gene pairs that are (a) null for both tissues, (b) null for tissue one and non-null for tissue 2, (c) non-null for tissue one and null for tissue 2, (d)non-null for both tissues. Note that π sums to 1. We constrain the hyperparameters $a_{j}\in [0,1]$ and $b_{j}\geq 1$ to encode the assumption that non-null SNP-gene pairs should have low p-values. Due to the large number of SNP-gene pairs tested, in practice we bin p-values on a regular 100×100 grid and use the bin counts to weight the likelihood. Finally we estimate the mutual information between the pair of tissues as:

(9) $$MI=\sum_{k=\left\{0,1\right\}}\;\sum_{j=\left\{0,1\right\}}\;\pi_{kj}\log\frac{\pi_{kj}}{\pi_{k}\pi_{j}^{\prime }}$$

where $\pi_{k}=\sum_{j=\{0,1\}}\;\pi_{kj}$ and ${\pi^{\prime }}_{j}=\sum_{k=\{0,1\}}\;\pi_{kj}$ are marginal probabilities. This approach explicitly estimates the proportion of null tests in tissue 1 ($\pi_{0}$) and tissue 2 (${\pi^{\prime }}_{0}$) as well as the proportion of tests that are non-null in both ($\pi_{11}$). This approach both controls for power in both tissues and negates the need to choose arbitrary significance thresholds.

### Splicing analysis

We ran LeafCutter v0.2.6_dev (using default settings) which allows joint differential intron excision testing across more than two conditions. For each Alternative Splicing Cluster (ASC) LeafCutter fits a set of PercentSplicedIn probability vectors $\psi_{c}$, across detected splice junctions i, at each concentration c. For ASCs determined to be significantly (5% FDR) differential spliced across concentrations, we calculated the entropy $h_{c}=-\sum_{i}\;\psi_{ci}\log\psi_{ci}$ at each concentration c. We normalized these profiles as $\tilde{h_{c}}=h_{c}/\bar{h_{c}}$ and clustered these profiles, using KMeans_rcpp as above.

To investigate the relative usage of cryptic splice sites we first determined the set of 7792 splice junctions that (a) fell in ASCs determined to be significantly differentially spliced (5% FDR) and (b) had $\max_{c}\psi_{ci}-\min_{c}\psi_{ci}>0.1$. We obtained normalized intron excision rates by subtracting the per intron mean and dividing by the per intron standard deviation. These ψ profiles were clustered using KMeans_rcpp. Cryptic splice site usage was determined by considering all exons in Gencode v26 and ignoring transcript structure (i.e. a junction spanning two splice sites used but only in different transcripts would still be considered ‘annotated’).

For (response) splicing QTL we calculated within ASC intron excision ψ with pseudocount of 0.5, and set entries with 0 denominator (no reads for that ASC in that sample) to the mean across all other samples. These values were then (1) z-score normalized across samples and (2) quantile normalized to a normal across introns. QTL mapping was then performed using suez considering each intron as a ‘gene’.

### Modeling cardiac troponin level

We assessed the proportion of variance in cardiac troponin explained by gene expression response. Let $y_{ci}$ represent the troponin level measured in individual i at doxorubicin concentration c, normalized to have 0 mean and variance 1 across individuals at each concentration. Let $x_{cig}$ be the expression of gene g (in individual i at concentration c), z-score normalized across samples. We consider the linear model

(10) $$y_{ci}=\sum_{g}\;\beta_{g}x_{cig}+\epsilon_{ci}$$

where $\epsilon_{ci}∼N(0,\sigma_{\epsilon }^{2})$ is noise and the coefficients $\beta_{g}$ are given a prior $N(0,\sigma_{\beta }^{2}/G)$ where G=12,317 is the number of genes in the analysis. Integrating over $\beta_{g}$ we have

(11) $$y_{:}∼N\left(0,\sigma_{\beta }^{2}\frac{1}{G}\sum_{g}x_{:g}x_{:g}^{T}+\sigma_{\epsilon }^{2}I\right)$$

We optimize this model wrt $\sigma_{\beta }$ and $\sigma_{\epsilon }$ to obtain an estimate $\rho =\sigma_{\beta }^{2}/(\sigma_{\beta }^{2}+\sigma_{\epsilon }^{2})$ of the percent variance of y explained by x. A Bayesian credible interval for ρ is obtained under this model using 8000 iterations of Hamiltonian Monte Carlo (with the first 4000 discarded as burnin) implemented using RStan (Carpenter et al., 2016) (v2.16.2).

For the transcriptome-wide association study for cardiac troponin levels we use the R package glmnet (v2.0–13) to build elastic-net predictors of gene expression for each gene, using 10-fold cross-validation to choose λ and α=0.5 which we found gave comparable performance to higher values. A single model was learnt per gene jointly across concentrations, including main effects for all SNPs within 100 kb of the gene TSS, main effects for doxorubicin dosage (encoded categorically) and interaction terms between each SNP and the dosage factor. The fitted values on the test-folds from the cross-validation are known as the ‘prevalidation’ response. To test which genes have a significant genetic component we tested (using analysis-of-variance) whether the observed expression was better predicted under a linear model including the prevalidation values and the dosage variable than by dosage alone. The prevalidated response for the 3840/12317 genes (1% FDR) genes that are predictable from genotype are then used to predict troponin level, normalized as for Equation 10, using leave-out-one-cross-validated lasso regression.

### Code and data availability

All the custom analysis scripts used for this project are available at https://github.com/davidaknowles/dox (Knowles and Blischak, 2017; copy archived at https://github.com/elifesciences-publications/dox). The suez response eQTL mapping R package is available at https://github.com/davidaknowles/suez (Knowles, 2017; copy archived at https://github.com/elifesciences-publications/suez). The following data are available as Supplementary Data: (1) differential expression cluster assignments, (2) significant (5% FDR) eQTLs and sQTLs, (3) differential splicing results, (4) levels of cardiac troponin and the predicted transcriptomic response. In addition to the Supplementary Data included with this paper additional results are hosted at http://web.stanford.edu/~dak33/dox/ and Dryad (doi:10.5061/dryad.r5t8d04) including (1) gene-by-sample matrix of RNA-seq quantification (log counts per million), (2) LeafCutter intron excision quantification (3) *p*-values for all tested eQTLs, reQTLs, sQTLs, and rsQTLs, (4) RARG variant response and marginal trans-eQTLs, (5) RIN, RNA concentration and other technical covariates, (6) embryoid body imaging for all iPSC lines. The RNA-seq FASTQ files will be added to the dbGaP database (Tryka et al., 2014) under dbGaP accession phs000185 (https://www.ncbi.nlm.nih.gov/projects/gap/cgi-bin/study.cgi?study_id=phs000185). The genotype data files cannot be shared because releasing genotype data from a subset of individuals in the pedigree would enable the reconstruction of genotypes of other members of the pedigree, which would violate the original protocol approved by the research ethics board (Livne et al., 2015). The summary statistics for the ACT GWAS were given to us by the authors of the studies (Schneider et al., 2017; Serie et al., 2017).

## Additional files

Supplementary file 1. Assignment of each gene’s response profile to differing doxorubicin concentrations into one of 6 clusters.

Supplementary file 2. HOMER2 pathway enrichment for each cluster from Supplementary file 1. Cluster: One of six clusters. TermID: Gene set ID. Term: gene set name. Odds_ratio: enrichment of this gene set in this cluster compared to background. p: p-value from hypergeometric test. p_adjust: Benjamini-Hochberg adjusted p-value. Overlap: number of genes shared by the gene set and the cluster. cluster_size: number of genes not in the cluster. Geneset_size: number of genes in the cluster. Total genes: total number of genes considered.

Supplementary file 3. HOMER2 promoter motif enrichment for each cluster from Supplementary file 1. cluster: One of six clusters. motif_info: full motif name including GEO source if applicable. motif: name of transcription factor. mphConsensus: consensus motif using IUPAC notation. odds_ratio: enrichment of motif in promoters of genes in this cluster compared to background. log_p: log p-value from hypergeometric test. p_adjust: Benjamini-Hochberg adjusted p-value. Overlap: number of genes in the cluster with this motif in their promoter. Background_overlap: number of genes not in the cluster with this motif in their promoter. cluster_size: number of genes not in the cluster. hbackground_size: number of genes not in the cluster.

Supplementary file 4. All significant (5% FDR) marginal effect eQTL. gene: Ensembl gene ID. chr: Chromosome. pos: eSNP position. RSID: Rapid Stain Identification Series of the eSNP. p: p from suez Bonferroni corrected for the number of SNPs tested for the gene. q: Benjamini-Hochberg adjusted p-value.

Supplementary file 5. All significant (5% FDR) response eQTL using total expression only. Columns are as for Supplementary file 4

Supplementary file 6. All significant (5% FDR) response eQTL mapped using total and allele specific expression. Columns are as for Supplementary file 4

Supplementary file 7. LeafCutter splicing clusters and their association with doxorubicin concentration. cluster: Cluster ID. p: p-value for Dirichlet-Multinomial GLM likelihood ratio test for differential splicing across doxorubicin concentrations. q: Benjamini-Hochberg adjusted p-value.

Supplementary file 8. LeafCutter estimates of Percent Spliced In (PSI) for each splice junction (intron) at each doxorubicin concentration.

Supplementary file 9. Splicing QTL mapped using LeafCutter and suez. Columns are as for Supplementary file 4 except that column one is the splice junction (intron) rather than the gene.

Supplementary file 10. Splicing response QTL mapped using LeafCutter and suez. Columns are as for Supplementary file 9.

Supplementary file 11. Cardiac troponin levels at different doxorubicin dosages. cell_line: internal cell line ID and replicate number. dbgap: individual ID in dbGaP. dosage: doxorubicin concentration. troponin: cardiac troponin level.

Supplementary file 12. Optimal lasso model predicting normalized troponin level from gene expression. Troponin level was normalized to be zero-mean and unit-variance across individuals at each concentration. gene: Ensembl gene id. hugo: HUGO approved gene symbol. beta: regression coefficient from lasso.

Supplementary file 13. Optimal lasso model predicting normalized troponin level from the component of gene expression predictable from genotype. Troponin level was normalized to be zero-mean and unit-variance across individuals at each concentration. gene: Ensembl gene id. hugo: HUGO approved gene symbol. beta: regression coefficient from lasso.

Supplementary file 14. Genes significantly differentially expressed with respect to troponin level at each doxorubicin concentration. conc: doxorubicin concentration. gene: Ensembl gene id. rho: Spearman correlation between gene expression and troponin level. p: Spearman correlation p-value. p_adjust: Benjamini-Hochberg adjusted p-value.

Supplementary file 15. Optimal lasso model predicting ACT risk from gene expression using previously published data (Burridge et al., 2016). gene: Ensembl gene id. beta: regression coefficient from lasso.

Supplementary file 16. Colocalization analysis of response-eQTL and ACT GWAS. gene: Ensembl gene id. snps: number of SNPs tested. PP.H0.abf: posterior probability of no association. PP.H1.abf: posterior probability of GWAS association only. PP.H2.abf posterior probability of reQTL only. PP.H3.abf: posterior probability of GWAS variant and reQTL being independent. PP.H4.abf: posterior probability of GWAS and reQTL colocalizing to the same variant.fa

Transparent reporting form

### Data availability

All the custom analysis scripts used for this project are available at https://github.com/davidaknowles/dox (Knowles and Blischak, 2017). The suez response eQTL mapping R package is available at https://github.com/davidaknowles/suez (Knowles, 2017). The following data are available as Supplementary Data: 1) differential expression cluster assignments, 2) significant (5% FDR) eQTLs and sQTLs, 3) differential splicing results, 4) levels of cardiac troponin and the predicted transcriptomic response. In addition to the Supplementary Data included with this paper, further results are hosted at Dryad (doi:10.5061/dryad.r5t8d04) including 1) gene-by-sample matrix of RNA-seq quantification (log counts per million), 2) LeafCutter intron excision quantification 3) p-values for all tested eQTLs, reQTLs, sQTLs, and rsQTLs, 4) RARG variant response and marginal trans-eQTLs, 5) RIN, RNA concentration and other technical covariates, 6) embryoid body imaging for all iPSC lines. The RNA-seq FASTQ files will be added to the dbGaP database (Tryka et al., 2014) under dbGaP accession phs000185 (https://www.ncbi.nlm.nih.gov/projects/gap/cgi-bin/study.cgi?study_id=phs000185). The genotype data files cannot be shared because releasing genotype data from a subset of individuals in the pedigree would enable the reconstruction of genotypes of other members of the pedigree, which would violate the original protocol approved by the research ethics board (Livne et al., 2015). The summary statistics for the ACT GWAS were given to us by the authors of the study (Schneider et al., 2016; Serie et al. 2017).

The following datasets were generated:

Knowles DACourtney K BurrowsJohn D BlischakCarole OberJonathan K PritchardYoav Gilad2018Expression and splicing quantification, eQTLs and sQTLshttp://dx.doi.org/10.5061/dryad.r5t8d04Available at Dryad Digital Repository under a CC0 Public Domain Dedication

Courtney K BurrowsKnowles DAJohn D BlischakKristen M PattersonCarole OberJonathan K PritchardYoav Gilad2018Genetic Studies in the Hutteriteshttps://www.ncbi.nlm.nih.gov/projects/gap/cgi-bin/study.cgi?study_id=phs000185Available at dbGaP via dbGaP Authorized Access (accession no. phs000185.v5.p1)
