# Supplementary material for: Determining the genetic basis of anthracycline-cardiotoxicity by molecular response QTL mapping in induced cardiomyocytes
Source: eLife. 2018 May 8;7:e33480. doi: 10.7554/eLife.33480 (PMC6010343; doi:10.7554/eLife.33480)
Supplement: Peer review history

# Decision letter

**DOI:** 10.7554/eLife.33480.049

**Affiliations:** Oxford UniversityUnited Kingdom

In the interests of transparency, eLife includes the editorial decision letter and accompanying author responses. A lightly edited version of the letter sent to the authors after peer review is shown, indicating the most substantive concerns; minor comments are not usually included.

Thank you for submitting your article "Determining the genetic basis of anthracycline-cardiotoxicity by response QTL mapping in induced cardiomyocytes" for consideration by *eLife*. Your article has been favorably evaluated by Mark McCarthy (Senior Editor) and three reviewers, one of whom is a member of our Board of Reviewing Editors. The following individual involved in review of your submission has agreed to reveal his identity: Douglas Sawyer (Reviewer #2).

The reviewers have discussed the reviews with one another and the Reviewing Editor has drafted this decision to help you prepare a revised submission.

Summary:

This work presents a detailed analysis of how IPSCs from healthy volunteers can be used to investigate the molecular and cellular processes involved in differential response to a widely used chemotherapy, which has a high rate of adverse effects. The work provides new insights into the molecular functions, such as splicing, disrupted by doxorubicin, and some evidence that genetic factors that influence responses in gene expression correlate with risk for adverse events. Although previous work has established the value of IPSCs in this context, this paper presents several important advances.

Essential revisions:

I have chosen to include the individual reviews as each provides some useful insight. However, there are a few key features that should be addressed when revising the manuscript. I do not expect a full response to every comment raised by each reviewer – just the three highlighted here.

1) The reviewers noted that the evidence linking genetic risk for adverse responses to the genetics of gene expression regulation is rather weak. There are two suggestions for how to advance this. One is to use allele-specific expression to add power to the genetic response in expression analysis (reviewer 3).

2) A second suggestion is to provide a breakdown of the genetic enrichment in the GWAS data by QTL type – see reviewer 1. Also to revisit some of the genes discussed earlier in the paper in the light of the GWAS data.

3) The work should include a more detailed characterisation of the state of the IPSC lines – see reviewer 3 (point 3). This is part of the QC process and important to include.

*Reviewer #1:*

This paper uses functional genomic and genetic analyses of iPSC cardiomyocytes to investigate the phenomenon of cardiomyocyte toxicity arising from anthracycline chemotherapy. Given the high frequency of this adverse effect and the widespread use of doxorubicin (and related compounds) this is an important problem, though as yet rather little understood. There has been quite a lot of previous and somewhat related work – notably Burridge et al., 2016. However, this paper goes beyond that by using iPSC from 45 healthy individuals (compared to the 3 with ACT and 3 without) and by integrating genetic data.

From my reading, I think the advances made by this paper are:

1) Replication of some of the Burridge et al. results about molecular functions and processes that are influenced by doxorubicin and their correlation with ACT response.

2) A much broader investigation of the dosage effects and variation among individuals. Ultimately, providing convincing evidence that the iPSC system provides a good model for ACT and that variation seen among iPSC lines correlates with ACT susceptibility in vivo.

3) Evidence to support the hypothesis that doxorubicin dysregulates gene splicing.

4) Evidence that there are a large number of genetic variants that affect expression responses to doxorubicin and that these show strong (but by no means complete) overlap with variants that affect gene expression in primary tissue.

5) Evidence that genetic variants that influence risk of ACT in patients are enriched for variants that also affect the expression of genes in response to doxorubicin.

Overall, there is a lot to admire in the paper. It is an extremely impressive and comprehensive use of functional genomics to probe the expression response to doxorubicin in cardiomyocytes. By focusing on variation among individuals, it provides compelling evidence that a strong stress response is a good biomarker for positive outcome. However, the primary focus of the paper concerns the genetic basis of this response – and the (strong and specific) claim being made is that understanding the genetic influences on doxorubicin response in iPSC cardiomyocytes provides biological insight into ACT susceptibility. Personally, I think this claim is rather weak for two reasons.

1) First, the Burridge et al. paper established the relevance of iPSC cardiomyocytes as a model for ACT, so the novelty of this paper rests on light being shed by the integration of genetic data on the iPSC lines. However, the majority of the genetic differences affecting doxorubicin response seem to be seen in GTEx tissues – with only a modest increase enrichment in heart compared to other tissues. The key question is whether reQTLs – i.e. those that are only visible through the doxorubicin treatment – are *more* relevant to ACT than general QTLs. Otherwise, to analyse the authors could have simply used the gene expression data from Burridge, the GWAS data and the GTEx data to arrive at similar conclusions. A concrete suggestion would be to stratify the enrichment in Figure 5B by general eQTL, cardiomyocyte (GTEx) eQTL and doxorubicin-specific reQTL.

2) Second, the enrichment reported in the GWAS data is really very weak – and certainly not strong enough to direct any future experiments. Put a different way – it doesn't appear to me that genes with strong doxorubicin reQTLs are particularly strong causal determinants of ACT risk. Conversely – I was surprised to find no further analysis of RARG – given that it is the only established GWS risk variant for ACT. While I am convinced that gene expression can be a useful biomarker for ACT, I don't think this paper establishes expression of any specific gene (or pathway) as causal for ACT susceptibility. I would like to see something like a Mendelian randomisation analysis of the response trait identified in Figure 4D to provide evidence to support a causal link.

In summary, this is a rather beautifully executed and analysed experiment, but the central claim – that genetic variation influencing gene responses to doxorubicin treatment in iPSC cardiomyocytes can be used to understand the biology of the phenomenon – is not convincing.

*Reviewer #2:*

The Investigators took an interesting approach to understand the effects of anthracyclines on the heart, using iPSCs differentiated into cardiomyocytes from a series of subjects to test differential response to doxorubicin. They present an incredibly rich dataset showing the complexity of anthracycline-induced transcriptional changes that vary among individuals. They compare their findings to published GWAS studies exploring the same questions, and present their findings in a clear and thoughtful manuscript. The figures are clear and communicative. This noninvasive approach to utilizing human cells to study this clinically important problem is interesting and potentially very valuable. If applied to a group of samples from patients with and without anthracycline cardiotoxicity, it would be even more interesting as the findings would be more likely interpretable as related to the clinical risk of anthracycline induced cardiotoxicity.

The investigators chose to use samples from a Hutterite population, presumably based upon how the concept that this homogeneous population is good for identifying single gene Mendelian disorders. The genetic basis for cardiotoxicity of anthracyclines is likely more complicated. This limitation should be acknowledged by the authors.

Experimentally the investigators chose a range of doxorubicin concentrations that are high relative to what is likely the exposure of the heart in vivo in the context of clinical doxorubicin use. Extending the concentration range to 0.1 or even 0.05 μm would be worthwhile, as others have shown that cell toxicity can occur in this range in cells in vitro.

The investigators collected serum for cardiac Troponin T measurements, looking for in vitro signs of cytotoxicity. However there may be other forms of toxicity to the heart that occur without myocyte cytolysis, including the changes in transcription and splicing as presented here, as well as effects on endothelial and mesenchymal stem cells. Along these lines, is there any consideration for changes in other cell types, as iPSC cultures are heterogeneous. These issues should be acknowledged by the authors.

ACT can arise years after chemotherapy, it would be interesting to see if the cells were treated and then analzyed at a later timepoint than 24 hours to see if there are further differences than the acute changes after a 24 hour exposure.

In the Discussion the authors mention fibrosis playing a role in ACT. While this has been shown in human populations (e.g. PMID 29106497) using MRI, it is a relatively minor effect. The authors may want to update the references used to support the statement about fibrosis, as well as acknowledge it remains unclear how mechanistic this is in ACT.

*Reviewer #3:*

This paper presents an analysis of interindividual variation in ips-derived cardiomyocyte gene expression following exposure to doxorubicin (dox).

The authors characterise differential expression and splicing patterns at different dox concentrations, and find good evidence that the in vitromodel they have developed represents a reasonably accurate simulation of ACT in vivo, with the activation of apoptosis and DNA damage repair pathways. They also map QTLs that modulate gene expression and splicing at different dox concentrations, and suggest that these are enriched for GWAS loci for ACT. The paper is well written, the figures are pretty clear and the analyses seem sound. The study is underpowered (n=45) and this places limitations on the conclusions that can be drawn, however, I don't find that they overstate their findings. I have the following major comments:

1) I was surprised that they didn't use allele-specific signals to boost power for association detection because the lead author has recently published a method for doing exactly this in the GxE setting (Knowles et al., 2017). I would like to see whether the GWAS / reQTL examples they highlight are supported by patterns of allele-specific expression, some discussion of why AS signals weren't used in the analysis and what impact this might have on their results.

2) They should try colocalisation of their eQTL (response and otherwise) with the ACT GWAS and report the appropriate summary stats (e.g. posterior probs of shared causality if using "coloc"), as this can be helpful for others who may want to follow up individual associations experimentally.

3) One issue with the use of IPSCs is that there can be substantial variation in the make-up of the cell populations created. I would like them to do a better job of characterising the composition and maturation status of the IPS CMs they have derived – there are existing markers that they can use to distinguishwhether they have made more atrial / ventricular-like CMs, and whether there is variation in CM maturity (for example in this review: http://www.sciencedirect.com/science/article/pii/S0167527317300517)

4) For comparison, they should compute the pi1 scores for their eQTLs across all GTEx tissues (not just heart, brain and LCLs).

# Reply: Author response

**DOI:** 10.7554/eLife.33480.050

Essential revisions:

I have chosen to include the individual reviews as each provides some useful insight. However, there are a few key features that should be addressed when revising the manuscript. I do not expect a full response to every comment raised by each reviewer – just the three highlighted here.

1) The reviewers noted that the evidence linking genetic risk for adverse responses to the genetics of gene expression regulation is rather weak. There are two suggestions for how to advance this. One is to use allele-specific expression to add power to the genetic response in expression analysis (reviewer 3).

We have now incorporated an extension of EAGLE/WASP to the repeated measurements scenario and combined this signal with that from using suez on total expression. This increases the number of response eQTL (at 5% FDR) from 376 to 447 and improves the observed enrichment with the GWAS data. This improvement in power is somewhat modest which we hypothesize is due to a) suez already being reasonably well powered in this setting and b) the somewhat low sequencing depth of our samples.

2) A second suggestion is to provide a breakdown of the genetic enrichment in the GWAS data by QTL type – see reviewer 1. Also to revisit some of the genes discussed earlier in the paper in the light of the GWAS data.

We have added an extensive break-down of the enrichment in low GWAS p-values by expression and splicing QTL modality: steady-state in three different GTEx tissues, baseline (no doxorubicin) in our ICs, main effects only, main + response, and finally response eQTLs alone. We see substantial enrichment for our response QTLs, more moderate enrichment for the main effect IC eQTLs and no enrichment at all in the GTEx eQTLs, lending credence to the idea that response QTL mapping is valuable. Mapping response eQTLs using both total and allelic expression further improves the observed enrichment in the GWAS data.

In addition we have included an investigation of the role of the RARG variant in our data. Only two individuals in our panel are heterozygous the RARG variant (the rest are homozygous reference) but suez is able to detect 1 marginal effect and 5 response trans-eQTLs which represent candidate downstream mediators of the effect of RARG on ACT risk.

3) The work should include a more detailed characterisation of the state of the IPSC lines – see reviewer 3 (point 3). This is part of the QC process and important to include.

We have added extensive data supporting the validity of our iPSC panel (Figures 1—figure supplements 1-4). This includes qPCR for the major pluripotency genes, gene expression profiling analysis using PluriTest, embryoid body staining to confirm the ability of all our iPSC lines to differentiate into all three germ layers (images included in Supplementary Data) and assessment of EBV integration/expression.

Reviewer #1:

[…] Overall, there is a lot to admire in the paper. It is an extremely impressive and comprehensive use of functional genomics to probe the expression response to doxorubicin in cardiomyocytes. By focusing on variation among individuals, it provides compelling evidence that a strong stress response is a good biomarker for positive outcome. However, the primary focus of the paper concerns the genetic basis of this response – and the (strong and specific) claim being made is that understanding the genetic influences on doxorubicin response in iPSC cardiomyocytes provides biological insight into ACT susceptibility. Personally, I think this claim is rather weak for two reasons.

1) First, the Burridge et al. paper established the relevance of iPSC cardiomyocytes as a model for ACT, so the novelty of this paper rests on light being shed by the integration of genetic data on the iPSC lines. However, the majority of the genetic differences affecting doxorubicin response seem to be seen in GTEx tissues – with only a modest increase enrichment in heart compared to other tissues. The key question is whether reQTLs – i.e. those that are only visible through the doxorubicin treatment – are more relevant to ACT than general QTLs. Otherwise, to analyse the authors could have simply used the gene expression data from Burridge, the GWAS data and the GTEx data to arrive at similar conclusions. A concrete suggestion would be to stratify the enrichment in Figure 5B by general eQTL, cardiomyocyte (GTEx) eQTL and doxorubicin-specific reQTL.

We have added an extensive break-down of the enrichment in low GWAS p-values by expression and splicing QTL modality: please see response to editor above.

2) Second, the enrichment reported in the GWAS data is really very weak – and certainly not strong enough to direct any future experiments. Put a different way – it doesn't appear to me that genes with strong doxorubicin reQTLs are particularly strong causal determinants of ACT risk. Conversely – I was surprised to find no further analysis of RARG – given that it is the only established GWS risk variant for ACT. While I am convinced that gene expression can be a useful biomarker for ACT, I don't think this paper establishes expression of any specific gene (or pathway) as causal for ACT susceptibility. I would like to see something like a Mendelian randomisation analysis of the response trait identified in Figure 4D to provide evidence to support a causal link.

By using better powered meta-analyzed GWAS data, leveraging allelic-specific expression in the response eQTL mapping and breaking down the eQTLs into different classes we observe considerably stronger enrichment.

We have now added an investigation of the role of the RARG variant in our data – please see the response to the editor.

Unfortunately we don’t have sufficient sample size to perform a MR analysis for the transcriptomic response trait of Figure 4D directly. However, it is feasible to perform a Transcriptome-Wide Analysis Study (TWAS) for the effect of gene expression on troponin level. For each gene we built an elastic-net predictive model of expression at each doxorubicin concentration using SNPs within 100kb, with 10-fold cross- validation to choose the regularization parameters. The fitted predictions (known as the “pre-validation” values) then represent the genetically determined component of expression. We then used the 3840 genes with a statistically significant genetic component (at 1% FDR) to predict troponin level using leave-out-one-cross- validated (LOOCV) LASSO regression. 89% of the variance in troponin level can be explained by the genetic component of 102 genes. This analysis provides evidence for a causal link from genotype through gene expression to troponin level and highlights potential mediating genes. This TWAS approach here is analogous to two- stage least squares Mendelian Randomization.

In summary, this is a rather beautifully executed and analysed experiment, but the central claim – that genetic variation influencing gene responses to doxorubicin treatment in iPSC cardiomyocytes can be used to understand the biology of the phenomenon – is not convincing.

Reviewer #2:

The Investigators took an interesting approach to understand the effects of anthracyclines on the heart, using iPSCs differentiated into cardiomyocytes from a series of subjects to test differential response to doxorubicin. They present an incredibly rich dataset showing the complexity of anthracycline-induced transcriptional changes that vary among individuals. They compare their findings to published GWAS studies exploring the same questions, and present their findings in a clear and thoughtful manuscript. The figures are clear and communicative. This noninvasive approach to utilizing human cells to study this clinically important problem is interesting and potentially very valuable. If applied to a group of samples from patients with and without anthracycline cardiotoxicity, it would be even more interesting as the findings would be more likely interpretable as related to the clinical risk of anthracycline induced cardiotoxicity.

The investigators chose to use samples from a Hutterite population, presumably based upon how the concept that this homogeneous population is good for identifying single gene Mendelian disorders. The genetic basis for cardiotoxicity of anthracyclines is likely more complicated. This limitation should be acknowledged by the authors.

Previous work from the Ober and Gilad labs suggests that genetic mapping in the Hutterite population has value that extends beyond identifying Mendelian loci to complex traits, see e.g.

1) Yao, T.-C., Du, G., Han, L., Sun, Y., Hu, D., Yang, J.J., Mathias, R., Roth, L.A.,

Rafaels, N., Thompson, E.E., et al. (2014). Genome-wide association study of lung function phenotypes in a founder population. J Allergy Clin Immunol 133, 248-255.e241-210.

2) Cusanovich, D.A., Billstrand, C., Zhou, X., Chavarria, C., De Leon, S., Michelini, K., Pai, A.A., Ober, C., and Gilad, Y. (2012). The combination of a genome-wide association study of lymphocyte count and analysis of gene expression data reveals novel asthma candidate genes. Hum Mol Genet 21, 2111-2123.

3) Campbell, C.D., Mohajeri, K., Malig, M., Hormozdiari, F., Nelson, B., Du, G., Patterson, K.M., Eng, C., Torgerson, D.G., Hu, D., et al. (2014). Whole-genome sequencing of individuals from a founder population identifies candidate genes for asthma. PLoS ONE 9, e104396.

However, we agree that the Hutterites may not represent all genetic variation relevant to ACT, so we have included a new paragraph in the Discussion:

“We used a panel of Hutterites individual since this homogeneous population offers unique advantages for mapping genetic traits: exposure to a fairly uniform environment and less variable genetic background, despite still representing much of European diversity (Newman et al., 2004). We acknowledge however that the genetic basis of ACT susceptibility is clearly complex and some relevant genetic variation may not be well represented in this cohort.”

*Experimentally the investigators chose a range of doxorubicin concentrations that are high relative to what is likely the exposure of the heart* in vivo *in the context of clinical doxorubicin use. Extending the concentration range to 0.1 or even 0.05 μm would be worthwhile, as others have shown that cell toxicity can occur in this range in cells* in vitro.

We agree it could be valuable to extend to lower concentrations. The concentrations we used are actually considerably lower than we used in a pilot study. Unfortunately performing these experiments across all cell lines would represent an experimental effort beyond the scope of the current work. We will consider this suggestion for future studies.

*The investigators collected serum for cardiac Troponin T measurements, looking for* in vitro *signs of cytotoxicity. However there may be other forms of toxicity to the heart that occur without myocyte cytolysis, including the changes in transcription and splicing as presented here, as well as effects on endothelial and mesenchymal stem cells. Along these lines, is there any consideration for changes in other cell types, as iPSC cultures are heterogeneous. These issues should be acknowledged by the authors.*

We have added data showing that the average CM purity is 72% (minimum 41%). The fact that the most overlapping GTEx tissue with our eQTLs after the two heart tissues is fibroblasts suggests these may be represented. Unfortunately however it is challenging to tease apart potential cell-type specific effects with our bulk RNA-seq data. We have added a line to the Discussion to acknowledge this.

ACT can arise years after chemotherapy, it would be interesting to see if the cells were treated and then analzyed at a later timepoint than 24 hours to see if there are further differences than the acute changes after a 24 hour exposure.

As for the question of lower concentrations we agree this would be interesting but beyond our current scope.

In the Discussion the authors mention fibrosis playing a role in ACT. While this has been shown in human populations (e.g. PMID 29106497) using MRI, it is a relatively minor effect. The authors may want to update the references used to support the statement about fibrosis, as well as acknowledge it remains unclear how mechanistic this is in ACT.

Citation added and we have acknowledged the uncertainty in this finding.

Reviewer #3:

[…] I have the following major comments:

1) I was surprised that they didn't use allele-specific signals to boost power for association detection because the lead author has recently published a method for doing exactly this in the GxE setting (Knowles et al., 2017). I would like to see whether the GWAS / reQTL examples they highlight are supported by patterns of allele-specific expression, some discussion of why AS signals weren't used in the analysis and what impact this might have on their results.

We have now included an analysis jointly leveraging total and allele-specific expression, please see response to editor.

2) They should try colocalisation of their eQTL (response and otherwise) with the ACT GWAS and report the appropriate summary stats (e.g. posterior probs of shared causality if using "coloc"), as this can be helpful for others who may want to follow up individual associations experimentally.

We have now performed this analysis. While for 40/43 tested genes (with reQTL p<1e-5 and GWAS p<0.05) the posterior probability of colocalization is higher than for independent signals, both the GWAS and eQTL data are underpowered for this type of analysis and *coloc* estimates the highest posterior probability for no association for 32/43 genes.

3) One issue with the use of IPSCs is that there can be substantial variation in the make-up of the cell populations created. I would like them to do a better job of characterising the composition and maturation status of the IPS CMs they have derived – there are existing markers that they can use to distinguishwhether they have made more atrial / ventricular-like CMs, and whether there is variation in CM maturity (for example in this review: http://www.sciencedirect.com/science/article/pii/S0167527317300517)

We have added extensive characterization of the iPSCs and estimates of cardiomyocyte purity (see response to editor above).

4) For comparison, they should compute the pi1 scores for their eQTLs across all GTEx tissues (not just heart, brain and LCLs).

We’ve now done this across 48 tissues, please see Figure 2A and Figure 2—figure supplement 2 and Figure 5—figure supplement 5. We found that while there are a small number of tissues that have higher pi1 scores than the heart tissues (Figure 5—figure supplement 5) this is likely due to confounding with differential power across the GTEx tissues: an analysis designed to account for the differential power clearly shows the heart tissues having the strongest overlap (Figure 2—figure supplement 2) out of all the GTEx tissues.
